# Hydrogen-dependent dissimilatory nitrate reduction to ammonium enables growth of Campylobacterota isolates

**DOI:** 10.1093/ismejo/wraf092

**Published:** 2025-05-14

**Authors:** Hokwan Heo, Thanh Nguyen-Dinh, Man-Young Jung, Chris Greening, Sukhwan Yoon

**Affiliations:** Department of Civil and Environmental Engineering, Korea Advanced Institute of Science and Technology (KAIST), Daejeon 34141, Republic of Korea; Department of Microbiology, Biomedicine Discovery Institute, Monash University, Clayton, Victoria 3800, Australia; Interdisciplinary Graduate Program in Advance Convergence Technology and Science, Jeju National University, Jeju 63243, Republic of Korea; Department of Biology Education, Jeju National University, Jeju 63243, Republic of Korea; Department of Microbiology, Biomedicine Discovery Institute, Monash University, Clayton, Victoria 3800, Australia; Department of Civil and Environmental Engineering, Korea Advanced Institute of Science and Technology (KAIST), Daejeon 34141, Republic of Korea

**Keywords:** nitrogen cycling, dissimilatory nitrate reduction to ammonium (DNRA), respiratory hydrogen oxidation, hydrogenases, Campylobacterota

## Abstract

Dissimilatory nitrate reduction to ammonium (DNRA) is a key process used by diverse microorganisms in the global nitrogen cycle. For long, DNRA has been considered primarily as an organotrophic reaction, despite evidence that oxidation of inorganic electron donors also supports DNRA. Evidence of DNRA coupling with molecular hydrogen (H_2_) oxidation has been reported for several microbial isolates; however, the underlying physiology of the microbial process remains understudied. In this study, we report the isolation of two Campylobacterota strains, *Aliarcobacter butzleri* hDNRA1 and *Sulfurospirillum* sp. hDNRA2, which grow using H_2_ as the sole electron donor for DNRA, and physiological insights gained from a close examination of hydrogenotrophic DNRA in these isolates. In both batch and continuous cultures, DNRA *sensu stricto* (i.e. NO_3_^−^ reduction that includes stoichiometric NO_2_^−^-to-NH_4_^+^ reduction) was strictly dependent on the presence of H_2_ and exhibited stoichiometric coupling with H_2_ oxidation, indicating that electrons required for NO_2_^−^ reduction were unequivocally derived from H_2_. Successful chemostat incubation further demonstrated that hydrogenotrophic DNRA is viable under NO_3_^−^-limiting, H_2_-excess conditions. Genomic and transcriptomic analyses identified group 1b [NiFe]-hydrogenase and cytochrome *c*_552_ nitrite reductase as the key enzymes catalyzing hydrogenotrophic DNRA. In addition, metagenomic surveys revealed that bacteria capable of hydrogenotrophic DNRA are taxonomically diverse and abundant in various ecosystems, particularly in the vicinity of deep-sea hydrothermal vents. These findings, integrating physiological, genomic, and transcriptomic analyses, clarify that H_2_ can solely serve as a growth-supporting electron donor for DNRA and suggest potential significance of this microbial process in nitrogen- and hydrogen-related environmental biogeochemical cycles.

## Introduction

Nitrogen is an essential element for the sustenance and growth of all life forms [1]. Although indispensable as a nutrient, the excessive release of anthropogenic reactive nitrogen into the environment poses significant threats to global sustainability by exacerbating nutrient pollution and climate change [2–4]. The transformation of one nitrogen form to another, largely mediated by microorganisms, dictates its availability to primary producers, both when nitrogen abundance is beneficial (e.g. agricultural soils) and detrimental (e.g. eutrophic water bodies) [5, 6]. Microbial nitrogen-cycling processes, including nitrification (aerobic NH_4_^+^ oxidation to NO_3_^−^ via NO_2_^−^) and denitrification (anaerobic NO_3_^−^ reduction to NO, N_2_O, and N_2_) have been widely studied in this context, and also due to their significant contributions to the emission of the potent greenhouse gas N_2_O [7–9].

Another biological nitrogen-cycling process, dissimilatory nitrate reduction to ammonium (DNRA), despite also having been studied for decades, continues to offer discoveries that refine our understanding of the biogeochemical nitrogen cycle. Like denitrification, DNRA is an anaerobic respiratory pathway that reduces NO_3_^−^ via NO_2_^−^ for energy conservation [10]. What distinguishes DNRA from denitrification is the reaction step that reduces NO_2_^−^ to NH_4_^+^, typically catalyzed by cytochrome *c*_552_ nitrite reductases (NrfA) [11, 12]. The competition between DNRA and denitrification for NO_3_^−^/NO_2_^−^ has ecological significance, as DNRA retains nitrogen, thereby preventing or alleviating nitrogen loss via denitrification [5, 11]. Previous studies have mainly explained this competition through the C:N ratio (i.e. the ratio of organic electron donors to nitrogenous electron acceptors), assuming that DNRA is coupled to the oxidation of organic electron donors [13, 14]. For decades, DNRA has been hypothesized to be favored in environments with high C:N ratios due to its higher energy yield per electron acceptor, as supported by numerous field and laboratory observations [13–17].

Despite the limited physiological data available in the literature, DNRA has also been reported to couple with the oxidation of inorganic electron donors. Reduced sulfur compounds, namely, S^0^ and H_2_S/HS^−^/S^2−^, have been proposed as electron donors for chemolithotrophic DNRA, explaining the elevated DNRA activity and/or *nrfA* detection in sulfur-rich, highly reduced environments [18–21]. The coupling of DNRA with Fe^2+^ oxidation has also been suggested based on observations from cable bacteria and anammox [22, 23]. Chemolithotrophic DNRA has been observed in several axenic cultures, such as *Desulfurivibrio alkaliphilus*, using sulfide as an electron donor; however, current understanding remains limited, and definitive conclusions about its ecological implications are elusive [24, 25]. Particularly puzzling is the paucity of physiological investigations into DNRA coupled with H_2_ oxidation, despite the fact that this reaction is highly exergonic (Equations 1 and 2), H_2_ is a ubiquitous electron donor, and respiratory hydrogenases are widely distributed across diverse ecosystems [26–28].


(1) \begin{equation*} {{\mathrm{NO}}_3}^{-}+2{\mathrm{H}}^{+}+4{\mathrm{H}}_2\to{{\mathrm{NH}}_4}^{+}+3{\mathrm{H}}_2\mathrm{O}\ \left(\Delta{G}^{0\hbox{'}}=-74.95\ \mathrm{kJ}/\mathrm{mol}\ {\mathrm{e}}^{-}\right) \end{equation*}

(2) \begin{equation*} {{\mathrm{NO}}_2}^{-}+2{\mathrm{H}}^{+}+3{\mathrm{H}}_2\to{{\mathrm{NH}}_4}^{+}+2{\mathrm{H}}_2\mathrm{O}\ \left(\Delta{G}^{0\hbox{'}}=-72.73\ \mathrm{kJ}/\mathrm{mol}\ {\mathrm{e}}^{-}\right) \end{equation*}

Although a number of studies have provided evidence of NO_3_^−^ reduction to NH_4_^+^ in the presence of H_2_ in bacterial isolates, evidence of exclusive dependence on H_2_ as the growth-supporting electron donor has been lacking. Most, if not all, of these experiments included large amounts of organic growth supplements that could serve as primary or supplementary electron donors [29–32]. Mass balance calculations were omitted, possibly due to interference from these electron-rich additives. Furthermore, most deep-sea isolates reported to exhibit DNRA phenotype in H_2_ presence lacked identified ammonium-forming nitrite reductase [33]. Here, we report two newly isolated Campylobacterota strains capable of growth on hydrogenotrophic DNRA. By integrating physiological, genomic, and transcriptomic analyses, we confirmed that DNRA in these isolates is tightly redox-coupled to H_2_ oxidation and suggest the key genes involved in this process. Furthermore, data mining of the metagenome-assembled genomes (MAGs) within the Genome Taxonomy Database (GTDB) revealed that the genomic potential for hydrogenotrophic DNRA is widespread among microorganisms affiliated with the phylum Campylobacterota found across diverse environments, and that *Sulfurospirillum* spp., which exhibit high genomic similarity to one of these isolates, are particularly abundant in the vicinity of deep-sea hydrothermal vents.

## Materials and methods

### Culture medium and growth conditions

The defined medium used for enrichment, isolation, and routine cultivation contained, per liter of deionized water: 4.76 mmol NaCl, 0.47 mmol CaCl_2_·2H_2_O, 0.24 mmol MgCl_2_·6H_2_O, 0.2 mmol NH_4_Cl, and 1 ml trace mineral solution (Supplementary Table S1). For batch incubations, 100 ml medium was prepared in 590-ml glass bottles sealed with bromobutyl-rubber stoppers. After autoclaving, the medium was supplemented with filter-sterilized 5 mM piperazine-N,N′-bis(2-ethanesulfonic acid) (PIPES; pH 7.0), Wolin’s vitamin solution, and 0.15 mM KH_2_PO_4_. The bottles were equilibrated with a H_2_/CO_2_/N_2_ (5:5:90) mixed gas (World-Enersys, Daejeon, South Korea), unless otherwise specified. Sodium acetate and KNO_3_ were added from autoclaved stock solutions. All cultures were incubated in the dark at 25°C with shaking at 200 rpm. Agar plates were prepared by adding 1.5% (w/v) Bacto agar (BD, Franklin Lakes, NJ). Inoculated agar plates (and open aqueous cultures) were incubated in an anaerobic chamber (Coy Laboratory Products, Grass Lake, MI) with H_2_/CO_2_/N_2_ (4:5:91) atmosphere.

### Isolation, screening, and cultivation

Activated sludge was sampled from the anoxic segment of a municipal wastewater treatment plant in Daejeon, South Korea (36°23′09.4"N, 127°24′28.5"E) on 26 October 2020. The initial goal was to isolate DNRA-catalyzing microorganisms that use acetate as their sole electron donor. In the anaerobic chamber, 20 ml of activated sludge was diluted into 200 ml medium containing 10 mM acetate and 2 mM NO_3_^−^ in a loosely capped 590-ml glass bottle. Cycloheximide was added to a concentration of 0.01% (w/v) to inhibit fungal growth, and the culture was stirred at 500 rpm in the dark. Every two weeks, 20 ml of the enrichment was transferred to fresh medium. After three transfers, the enrichment was serially diluted and spread onto agar plates containing 20 mM acetate and 10 mM NO_3_^−^. Single colonies were screened for the DNRA phenotype [16].

### Discovery, verification, and physiological characterization of hydrogenotrophic DNRA

A 200-μl suspension from a single colony was inoculated into 100 ml medium containing 10 mM acetate and 2 mM NO_3_^−^ and equilibrated with N_2_. After initial attempts to reproduce DNRA activity failed, H_2_ was tested as a potential electron donor. Batch cultures were prepared with the following conditions: (i) H_2_/CO_2_/N_2_ (5:5:90) with 10 mM acetate, (ii) H_2_/N_2_ (5:95) with 10 mM acetate, (iii) H_2_/CO_2_/N_2_ (5:5:90) without organic carbon, and (iv) H_2_-free control (CO_2_/N_2_ 5:95) with 10 mM acetate. All cultures contained 2 mM NO_3_^−^. Headspace H_2_ and N_2_O, dissolved NO_3_^−^, NO_2_^−^, NH_4_^+^, and acetate concentrations, and cell density (OD_600_) were monitored, and pH was measured before and after incubation. The two isolates that exhibited growth and consumed H_2_ and NO_3_^−^, producing NH_4_^+^, were taxonomically identified using Sanger sequencing of 16S rRNA genes amplified with the 27F/1492R primer set.

Coupling of DNRA with H_2_ oxidation of the two isolates, now termed *Aliarcobacter butzleri* hDNRA1 and *Sulfurospirillum* sp. hDNRA2, was further examined using intermittent H_2_ starvation experiments. Cultures were prepared with 2 mM NO_3_^−^ and a CO_2_/N_2_ (5:95) headspace. Initial acetate concentrations were 1 mM for *A. butzleri* hDNRA1 and 0.1 mM for *Sulfurospirillum* sp. hDNRA2. A limiting amount of H_2_ (200 μmol per bottle) was injected at the start, and NO_3_^−^, NO_2_^−^, NH_4_^+^, and headspace H_2_ concentrations were monitored. Additional H_2_ (200 μmol) was injected after verifying cessation of NO_3_^−^/NO_2_^−^ reduction following H_2_ depletion.

### Kinetics of anaerobic H_2_ oxidation

Whole-cell Michaelis–Menten kinetics were measured to examine H_2_ affinities of the two isolates. Cultures were grown in medium with 1 mM acetate and 2 mM NO_3_^−^ and a H_2_/CO_2_/N_2_ (5:5:90) headspace. After NO_3_^−^/ NO_2_^−^ depletion, bottles underwent three vacuum-pressurization cycles with N_2_ gas, followed by the addition of 10 mM NO_2_^−^ and H_2_ (100–100 000 ppmv) generated with a PG-H_2_ Plus Hydrogen Generator (PerkinElmer, Waltham, MA). Initial H_2_ consumption rates were calculated from headspace concentrations measured with 20-minute intervals. Dry biomass was quantified from triplicate 100-ml cultures grown under identical conditions [34]. *V*_max(app)_ and *K*_*m*(app)_ values were calculated from the dataset obtained from triplicate experiments via nonlinear least-squares regression analysis using GraphPad Prism v9.5.1.

### Continuous incubation on hydrogenotrophic DNRA

A chemostat reactor (1.14-l glass vessel, 600-ml working volume) was stirred at 650 rpm and operated at a dilution rate of 0.025 h^−1^ with medium containing 1 mM acetate and 2 mM NO_3_^−^, maintained anoxic with N_2_ flushing (Supplementary Fig. S1). Incubation began as a batch culture, continuously supplied with a stream of H_2_/CO_2_/N_2_ (0.5:0.5:99) mixed gas at 30 ml min^−1^, and the reactor was transitioned to chemostat operation after NO_3_^−^/NO_2_^−^ depletion. NO_3_^−^, NO_2_^−^, and NH_4_^+^ concentrations and cell density were monitored. For *Sulfurospirillum* sp. hDNRA2, gas mixing ratios and flow rates were adjusted during incubation. Once nitrogen species concentrations stabilized, H_2_ was excluded from the gas stream to assess DNRA dependency on H_2_.

### Analytical methods

H_2_ concentrations were measured using an 8890 gas chromatograph with a thermal conductivity detector and MolSieve 5 Å and Porapak Q columns (Agilent Technologies, Santa Clara, CA), using Ar (≥99.999%) as the carrier and reference gas. N_2_O concentrations were determined with another 8890 gas chromatograph equipped with a micro-electron capture detector and a HP-PLOT Q column, using He (≥99.999%) as the carrier gas and CH_4_/Ar (5:95) as the make-up gas. Dissolved H_2_ and N_2_O concentrations were calculated from headspace concentrations using dimensionless Henry’s constants (aqueous/gaseous; 25°C) of 0.0193 and 0.595, respectively [35]. NO_3_^−^, NO_2_^−^, and NH_4_^+^ concentrations were measured colorimetrically, and acetate concentration were analyzed using a Prominence HPLC system (Shimadzu, Kyoto, Japan) [36, 37].

### Genome sequencing

Genomic DNA was extracted from batch cultures using the DNeasy Blood & Tissue kit (Qiagen, Germany). A hybrid genome sequencing approach was employed, combining HiSeq (Illumina, San Diego, CA) and MinION (Oxford Nanopore Technologies, Oxford, UK) sequencing. A paired-end sequencing library, prepared using the TruSeq DNA PCR-Free kit (Illumina), was sequenced at Macrogen (Seoul, South Korea) with a 4-Gb throughput. Raw reads were trimmed using Trimmomatic v0.39 and assembled using SPAdes v3.15.3 [38, 39]. Long-read sequencing was performed on a MinION sequencer using a R9.4.1 flow cell with libraries prepared using the Ligation Sequencing kit (Oxford Nanopore Technologies). Raw sequences were processed with Guppy v6.2.11 (Oxford Nanopore Technologies) and Porechop v0.2.4 [40]. A short-read-first hybrid assembly was constructed using Unicycler v0.4.8 and annotated using Prokka v1.14.5 [41, 42].

### Transcriptome sequencing

Triplicate cultures were grown in a 5-l glass bottle containing 1 l medium supplemented with 1 mM acetate and 2 mM NO_3_^−^ and a H_2_/CO_2_/N_2_ (5:5:90) headspace. Cultures of *A. butzleri* hDNRA1 grown without H_2_ and *Sulfurillosprillum* sp. hDNRA2 grown without H_2_ (5:95 CO_2_/N_2_ gas in the headspace) but with 5 mM formate were also prepared. Cell density and NO_3_^−^/NO_2_^−^ concentrations were monitored to ensure that the samples were collected during the exponential phase (Supplementary Fig. S2). The entire batch was filtered through a 0.22-μm Sterivex filter unit (Merck Millipore, Germany). Subsequently, 10 ml RNAprotect bacteria reagent (Qiagen) was passed through the filter, which was then stored at −80°C. After thawing on ice, the membrane filter was disrupted using acid-washed glass beads. The crude lysate was processed with RNeasy Mini kit, RNase-Free DNase, and RNeasy MinElute Cleanup kit (Qiagen). Following treatment with the Ribo-Zero Plus rRNA Depletion kit, the RNA-Seq library was prepared using the Stranded Total RNA Prep kit (Illumina). Paired-end sequencing was performed on a NovaSeq 6000 platform (Illumina) at Macrogen with a 5-Gb throughput. The biological triplicates were processed and sequenced independently.

### Transcriptome analyses

Raw reads were quality-trimmed using Trimmomatic v0.36 and mapped to the genomes using Bowtie2 v2.5.1 [43]. The alignments were converted to binary alignment map (BAM) format and sorted using SAMtools v1.17 [44]. Read counts were computed using featureCounts v2.0.6 [45]. Equation 3 was used to calculate fragments per kilobase of transcript per million reads mapped (FPKM) values.


(3) \begin{equation*} {\mathrm{FPKM}}_i=\frac{C_i\times{10}^9}{N\times{L}_i} \end{equation*}

where *C_i_* is the number of fragments mapped to the gene of interest, *N* is the total number of mapped fragments, and *L_i_* is the gene length (bases). The transcription data were analyzed using the “*DESeq2*” package v1.42.0, with the biological triplicate dataset as input [46]. Genes with |log_2_(fold change)| > 1 and an adjusted *P* value < .05 (Benjamini–Hochberg method) from pairwise comparisons between two incubation conditions were considered differentially expressed.

### Analyses of metagenome-assembled genomes from GTDB

GTDB R09-RS220A was searched for MAGs affiliated with the Campylobacterota phylum, using selection criteria of ≥ 90% completeness and ≤ 5% contamination [47]. Protein-coding genes were predicted using Prodigal v2.6.3 [48]. The hidden Markov models (HMMs) for NapA (KEGG Orthology ID: K02567) and NrfA (K03385) were downloaded from KofamKOALA [49]. The HMM for HynB, the large catalytic subunit of the group 1b [NiFe]-hydrogenase, was constructed from a MAFFT (v7.526) alignment of 52 Epsilonproteobacteria HynB sequences downloaded from HydDB (updated 2 September 2018), using *hmmbuild* command in HMMER v3.4 [50–52]. The MAGs were screened for the presence of all three target genes using *hmmsearch*, applying an *E*-value threshold of 1e-20 for NrfA and NapA, and 1e-150 for HynB. The HMMER-screened MAGs were annotated with Prokka v1.14.6 [42].

For MAGs with traceable source metagenomic data, relative abundances were computed by mapping the raw metagenomic reads onto the contigs constituting the MAGs. The raw metagenomic data were downloaded from the NCBI Sequence Read Archive (accessed 14 October 2024; Supplementary Table S2). Initial quality assessment was conducted using FastQC v0.12.1 [53]. The reads processed with Trimmomatic v0.39 were mapped to the indexed MAGs, using the *bwa mem* command in BWA v0.7.17 [38, 54]. The mapped reads were processed with SAMtools v1.17 [44]. The percentage of metagenomic reads mapped to a MAG represented the relative abundance of the corresponding microorganism.

### Statistical analysis

All statistical analyses were performed using R v4.3.1 (www.r-project.org). Statistical significance was determined using Student’s *t*-tests: paired *t*-test for comparison between two time points within a time-series dataset, and unpaired *t*-tests for comparison across two different treatments.

## Results

### New Campylobacterota isolates mediate hydrogenotrophic DNRA

**Figure 1:**
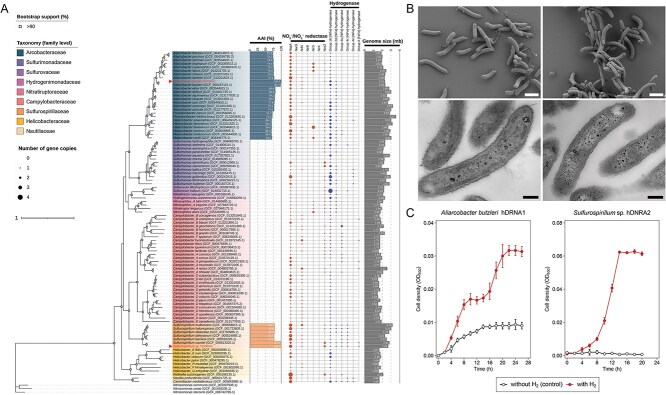
Phylogenetic affiliation, cellular morphology, and growth characteristics of *A. butzleri* hDNRA1 and *Sulfurospirillum* sp. hDNRA2 during hydrogenotrophic DNRA. (A) Maximum-likelihood phylogenetic tree of 92 Campylobacterota genomes, including the two isolates, constructed using IQ-TREE v2.3.6. The tree is based on the alignment of the concatenated amino acid sequences from 120 single-copy bacterial marker genes identified with GTDB-Tk v2.4.0 (release220). Three *Nitrosomonas* spp. genomes served as the outgroup. Branch support values were derived from 1000 ultrafast bootstrap replicates and the SH-aLRT single-branch test; bifurcations with both support values > 80% are marked with filled black circles. A grid plot to the right of the tree visualizes the inventories of functional genes encoding dissimilatory NO_3_^−^/NO_2_^−^ reductases and hydrogenases. (B) SEM (top) and TEM (bottom) images of *A. butzleri* hDNRA1 (left) and *Sulfurospirillum* sp. hDNRA2 (right). Scale bars in the micrographs represent 1 μm and 0.2 μm for SEM and TEM images, respectively. (C) Batch-culture growth curves of *A. butzleri* hDNRA1 and *Sulfurospirillum* sp. hDNRA2, grown with H_2_, acetate, and NO_3_^−^. Each data point represents the mean of three biological replicates (*n* = 3), with error bars indicating standard deviations.

We isolated two bacteria capable of coupling NO_3_^−^/NO_2_^−^-to-NH_4_^+^ reduction with H_2_ oxidation. Genomic analyses revealed their phylogenetic affiliation with the genera *Aliarcobacter* and *Sulfurospirillum*, both belonging to the phylum Campylobacterota (Fig.  1A). Their closest type strains were *A. butzleri* RM4018 (99.9% 16S rRNA gene sequence identity; digital DNA–DNA hybridization score of 76.1% using the formula *d_4_*) and *Sulfurospirillum cavolei* NBRC 109482 (99.5% and 67.0%, respectively). Thus, the two new isolates are designated as *A. butzleri* hDNRA1 and *Sulfurospirillum* sp. hDNRA2. Both isolates exhibited a curved rod-shaped morphology with approximate dimensions of 1.5–2.5 μm × 0.3–0.4 μm (Fig.  1B). Only *A. butzleri* hDNRA1 possessed a polar flagellum-like appendage.

**Figure 2:**
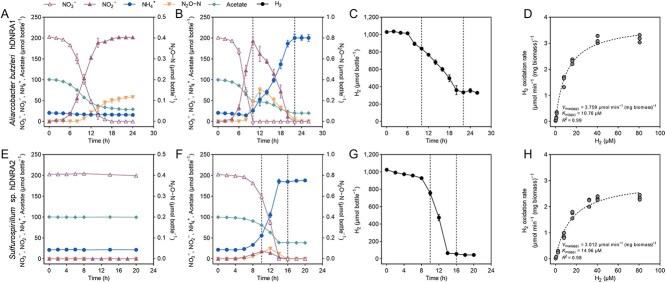
Hydrogenotrophic DNRA and H_2_ oxidation kinetics in *A*. *butzleri* hDNRA1 and *Sulfurospirillum* sp. hDNRA2. (A, B, E, F) Reductive transformation of NO_3_^−^ (2 mM; 200 μmol per bottle) was monitored in batch cultures under two conditions: in the absence (A, E) and presence (B, F) of 5% (v/v) H_2_ in the headspace. Concentrations of NO_3_^−^, NO_2_^−^, NH_4_^+^, and acetate were monitored, with dotted vertical lines indicating the NO_2_^−^ peak (t=10 h) and the depletion of NO_3_^−^/NO_2_^−^ (t=16 h). (C, G) H_2_ consumption during the experiments (B, F) was tracked over time to assess its role as an electron donor for DNRA. Each data point represents the mean of three biological replicates (*n* = 3), with error bars indicating standard deviations. (D, H) Michaelis–Menten kinetics of H_2_ oxidation were evaluated for whole cells pregrown on hydrogenotrophic DNRA. Initial H_2_ oxidation rates were calculated from batch incubations (*n* = 3) initiated with the indicated molar concentrations of dissolved H_2_ on the *x*-axis.

A series of batch experiments confirmed that H_2_ can serve as the sole electron donor for DNRA *sensu stricto* (i.e. NO_3_^−^ reduction that includes stoichiometric NH_4_^+^ production from NO_2_^−^) in these isolates. Despite the presence of acetate in the medium, which *A. butzleri* hDNRA1 could utilize as the electron donor for NO_3_^−^-to-NO_2_^−^ reduction, NO_2_^−^-to-NH_4_^+^ reduction required H_2_ (Figs  1C and 2A–C). In the cultures amended with H_2_ and acetate, *A. butzleri* hDNRA1 completely consumed 201 ± 1 μmol NO_3_^−^, of which 181 ± 2 μmol was converted to NH_4_^+^, exhibiting a diauxic growth. Oxidation of 670 ± 30 μmol H_2_, yielding 1340 ± 60 μmol e^−^, accounted for 84 ± 4% of the theoretical electron demand. *Sulfurospirillum* sp. hDNRA2 was unable to couple NO_3_^−^ reduction with acetate oxidation; therefore, H_2_ served as the sole electron donor (Fig. 2E). The electron yield from H_2_ oxidation (1910 ± 20 μmol e^−^) greatly exceeded the theoretical electron demand (1330 ± 10 μmol e^−^), suggesting that *Sulfurospirillum* sp. hDNRA2 allocated ~ 30% of the electrons from H_2_ oxidation for assimilation (Fig. 2F and G). Under identical NO_3_^−^-reducing conditions, *Sulfurospirillum* sp. hDNRA2 achieved significantly higher biomass (OD_600_ value of 0.064 ± 0.001 compared to 0.032 ± 0.002 of *A. butzleri* hDNRA1; *P* value < .05) (Fig.  1C).

Transient accumulation of NO_2_^−^ and N_2_O was observed in both isolates but was substantially higher in *A. butzleri* hDNRA1 cultures. Neither isolate exhibited significant growth on H_2_ without acetate, as evidenced by the lack of a significant change in OD_600_ value during incubation (*P* value > .05); however, *Sulfurospirillum* sp. hDNRA2 exhibited significant NH_4_^+^ production (*P* value < .05), which was sustained for > 72 h (Supplementary Fig. S3). *Aliarcobacter butzleri* hDNRA1 reduced NO_3_^−^ to NO_2_^−^ but not to NH_4_^+^.

### H_2_ oxidation is directly coupled to DNRA

**Figure 3:**
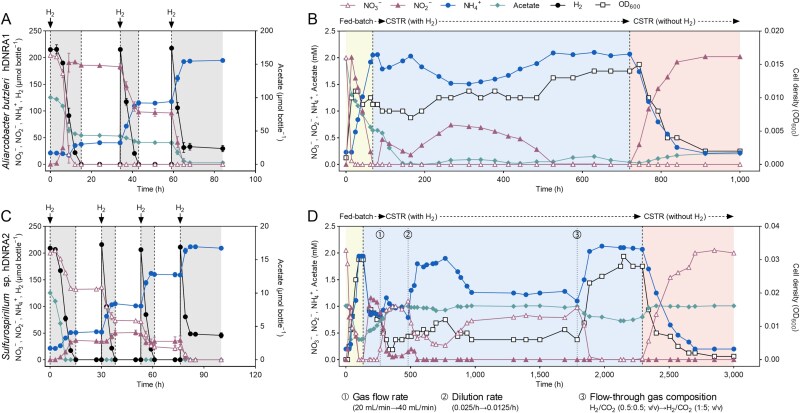
Coupling of H_2_ oxidation with DNRA in H_2_-limited batch cultures and chemostat reactor of *A*. *butzleri* hDNRA1 and *Sulfurospirillum* sp. hDNRA2. (A, B) Hydrogen-limited batch cultures were initiated with a stoichiometrically limiting amount of H_2_ (200 μmol bottle^−1^; H_2_-to-NO_3_^−^ molar ratio of 1) and replenished with H_2_ (200 μmol bottle^−1^) after its depletion (indicated by black arrows). The concentrations of NO_3_^−^, NO_2_^−^, NH_4_^+^, H_2_, and acetate were monitored until the complete depletion of NO_3_^−^/NO_2_^−^ and are presented as total amounts in the bottles. Each data point represents the mean of three biological replicates (*n* = 3), with error bars indicating standard deviations. (C, D) Hydrogenotrophic DNRA was demonstrated in lab-scale chemostat reactors. For *Sulfurospirillum* sp. hDNRA2, experimental conditions were optimized during incubation to achieve a steady state, with vertical dotted lines marking the time points of changes (details provided below the figure). At the end of the incubation, H_2_ was removed from the gas stream to evaluate its impact on DNRA activity.

The redox-coupling of DNRA *sensu stricto* to H_2_ oxidation was further verified through batch incubations with intermittent H_2_ starvation periods. *Aliarcobacter butzleri* hDNRA1 completely reduced 204 ± 4 μmol NO_3_^−^ prior to the first H_2_ starvation period, presumably utilizing both acetate and H_2_ as electron donors (Fig. 3A). The NO_2_^−^-to-NH_4_^+^ turnover halted immediately following H_2_ depletion. H_2_ injection at *t* = 34 and 59 h immediately resumed NO_2_^−^-to-NH_4_^+^ reduction. Acetate was consumed only when H_2_ was being consumed; however, a portion of electron from acetate may have been directed to NO_2_^−^ after repeated H_2_ starvations, as inferred from the observed discrepancy between the amounts of H_2_ oxidized (185 ± 6 μmol; 369 ± 13 μmol e^−^) and the NO_2_^−^ reduced to NH_4_^+^ (96 ± 6 μmol; 578 ± 37 μmol e^−^) beyond *t* = 59 h.

The experiment with *Sulfurospirillum* sp. hDNRA2, performed under identical conditions but with a reduced initial amount of acetate (10 μmol bottle^−1^), not only confirmed that H_2_ served as the sole electron donor for both NO_3_^−^-to-NO_2_^−^ and NO_2_^−^-to-NH_4_^+^ reduction but also demonstrated that these reductions could be sustained without organic carbon source for an extended period (Fig. 3C). Both reduction processes occurred simultaneously. The ratio of electron distribution to NO_3_^−^ and NO_2_^−^ from the initially added H_2_ was 0.70; however, this ratio decreased to 0.44 for H_2_ added at *t* = 30 h and further decreased to 0.28 for H_2_ added at *t* = 53 h, indicating a shift in electron allocation between NO_3_^−^ and NO_2_^−^. Even after acetate depletion at *t* = 9 h, DNRA activity was sustained for > 70 h, resulting in complete reduction of remaining NO_3_^−^ and NO_2_^−^ to NH_4_^+^.

The kinetics of H_2_ oxidation coupled to NO_2_^−^-to-NH_4_^+^ reduction by both isolates fit well to the Michaelis–Menten kinetics model (*R*^2^ > 0.97; Fig. 2D and H). The *K*_*m*(app)_ values were calculated to be 10.8 μM (95% confidence interval: 9.0–12.9) and 15.0 μM (95% confidence interval: 11.5–19.5) for *A. butzleri* hDNRA1 and *Sulfurospirillum* sp. hDNRA2, respectively. The *V*_max(app)_ values were 3.76 μmol min^−1^ (mg biomass)^−1^ (3.56–3.97) and 3.01 μmol min^−1^ (mg biomass)^−1^ (2.76–3.30), respectively. Therefore, the two isolates apparently share a comparable target H_2_ concentration range and maximum turnover rates.

### Hydrogenotrophic DNRA is sustained during NO_3_^−^-limiting continuous cultivation

The feasibility of establishing a NO_3_^−^-limiting steady-state culture with the two isolates was examined (Fig. 3B and D). The *A. butzleri* hDNRA1 chemostat, supplied with a stream of H_2_/CO_2_/N_2_ (0.5:0.5:99) mixed gas, reached a steady-state after 526 h, with no detectable NO_3_^−^ (<0.01 mM) and 2.0 ± 0.1 mM NH_4_^+^ in the steady-state culture. The steady-state NO_3_^−^ consumption and NH_4_^+^ production rates were 30.5 ± 0.6 and 28.1 ± 0.4 μmol h^−1^, respectively. Acetate was consumed at a rate of 13.7 ± 0.7 μmol h^−1^. The H_2_ concentrations in the influent and effluent gas streams were indistinguishable (*P* value > .05), consistent with stoichiometric calculations indicating H_2_ was supplied in excess (4018 μmol h^−1^ in the flow-through gas, compared to the theoretical demand of 245 μmol h^−1^, assuming H_2_ serves as the sole electron donor for NO_3_^−^-to-NH_4_^+^ reduction). After ~200 h of steady-state reactor operation, the gas supply was switched to exclude H_2_, and the NO_2_^−^ concentration in the reactor gradually increased. A new steady-state was established, at which the NO_2_^−^ concentration matched the NO_3_^−^ concentration in the influent, corroborating that DNRA *sensu stricto* was strictly coupled to H_2_ oxidation.

Establishing a continuous culture of *Sulfurospirillum* sp. hDNRA2 required additional optimization (Fig. 3D). The initial conditions failed to achieve the targeted steady state. Only after H_2_ and CO_2_ concentrations in the gas stream were increased to 1% and 5%, respectively, the reactor completely reduced 2 mM NO_3_^−^ to NH_4_^+^ at a rate of 15.65 ± 0.24 μmol h^−1^. During this steady-state operation, acetate was consumed at 1.73 ± 0.35 μmol h^−1^. The removal of H_2_ resulted in washout, confirming that the reduction of both NO_3_^−^ and NO_2_^−^ in *Sulfurospirillum* sp. hDNRA2 was coupled to H_2_ oxidation.

### Both strains encode hydrogenases and Nap and Nrf for hydrogenotrophic DNRA

**Figure 4:**
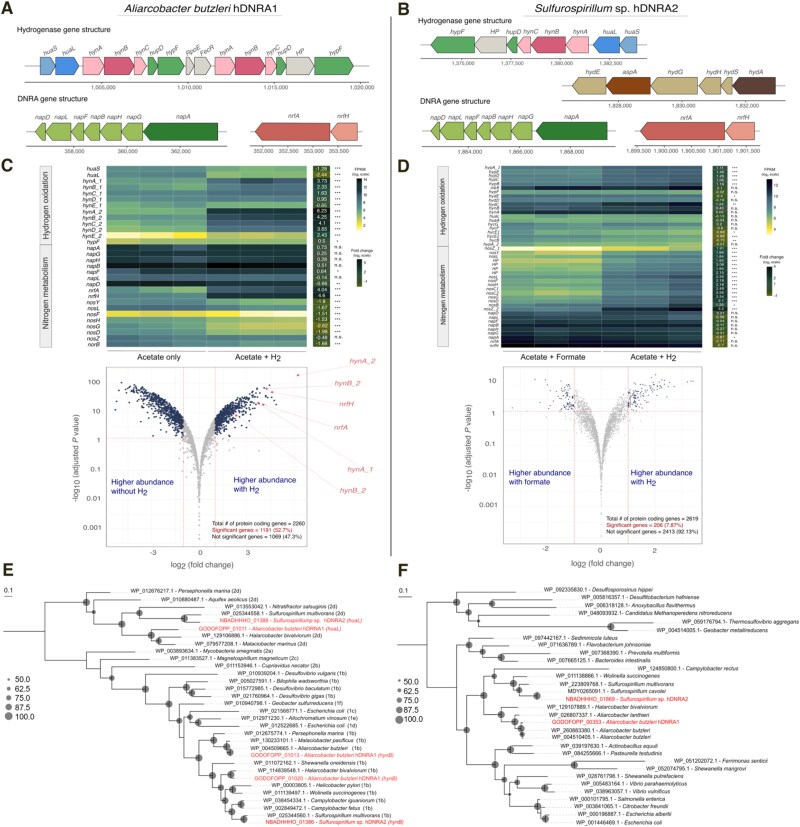
Genomic and transcriptomic insights into hydrogenotrophic DNRA in *A*. *butzleri* hDNRA1 and *Sulfurospirillum* sp. hDNRA2. (A, B) Gene clusters putatively associated with hydrogenotrophic DNRA identified from the complete genomes of *A*. *butzleri* hDNRA1 and *Sulfurospirillum* sp. hDNRA2. (C, D) Differential gene expression analyses of functional genes involved in hydrogen oxidation and nitrogen metabolism (top). Genome-wide transcriptional changes under H_2_-free (acetate and formate used as alternate electron donors for *A. butzleri* hDNRA1 and *Sulfurospirillum* sp. hDNRA2, respectively) versus H_2_-supplemented conditions are shown in volcano plots (bottom). Datasets from three biological replicates (*n* = 3) are presented for each condition. Maximum-likelihood phylogenetic trees generated with the amino acid sequences of the catalytic subunits of the group 1b [NiFe]- and group 2d [NiFe]-hydrogenases (E), and cytochrome *c*_552_ nitrite reductase (F) are presented to illustrate the phylogenetic positioning of these key functional genes in the two hydrogenotrophic DNRA isolates.

Closed genomes of *A. butzleri* hDNRA1 and *Sulfurospirillum* sp. hDNRA2 were obtained through hybrid assembly of short-read and long-read data (Supplementary Table S3). Both strains possess a *nap* cluster (*napAGHBFLD*) and a *nrf* cluster (*nrfAH*), encoding a respiratory periplasmic nitrate reductase and a cytochrome *c*_552_ nitrite reductase, respectively (Fig. 4A and Supplementary Table S4). The *nrf* cluster is immediately downstream of the *nap* cluster in *A. butzleri* hDNRA1, and in proximity (~30 kb apart) in *Sulfurospirillum* sp. hDNRA2, corroborating their functional relatedness. Although neither genome contains *nirK* or *nirS*, confirming their inability to denitrify, both genomes harbor clade II *nosZ* genes encoding the catalytic subunit of nitrous oxide reductase.

Structural and maturation genes for multiple hydrogenases were identified in the genomes. Both bacteria co-encode a group 1b [NiFe]-hydrogenase (*hynAB*) and a group 2d [NiFe]-hydrogenase (*huaSL*). In addition, in *A. butzleri* hDNRA1, another set of *hynAB* with 51.2% amino acid identity was identified nearby. Both isolates also possessed the *hypABCDEF* genes required for the maturation of [NiFe]-hydrogenases. *Sulfurospirillum* sp. hDNRA2 harbored groups 4a and 4c [NiFe]-hydrogenases that couple the oxidation of formate and carbon monoxide to H_2_ generation from protons, as well as a group A [FeFe]-hydrogenase flanked by H-cluster maturation assembly genes (*hydGEF*) and aspartate ammonia-lyase (*aspA*) (Supplementary Table S5). This hydrogenase inventory suggests that this strain can mediate a range of H_2_ oxidation and production, in addition to hydrogenotrophic DNRA. This strain also encodes formate dehydrogenase N (*fdnG*) and formate dehydrogenase H (*fdhF*), which may explain its ability to utilize formate as the source of electrons for NO_3_^−^ respiration (Supplementary Fig. S4).

The capacity for mixotrophic growth, specifically biomass production through carbon acquisition from acetate and CO_2_, was evident from the *Sulfurospirillum* sp. hDNRA2 genome (Supplementary Fig. S5). Acetate assimilation likely depends on acetate kinase and phosphate acetyltransferase, encoded by the *ackA* and *pta* genes, respectively. Acetyl-CoA may also be generated by the activity of acetate-CoA ligase, encoded by the *acs* gene. The reductive tricarboxylic acid (rTCA) cycle is the most likely pathway employed by *Sulfurospirillum* sp. hDNRA2 if it assimilates CO_2_, as many of the genes encoding this pathway, including those for 2-oxoglutarate synthase, isocitrate dehydrogenase, pyruvate carboxylase, succinate-CoA ligase (ADP-forming), and pyruvate synthase are present in the genome. However, two key genes in the conventional rTCA cycle, encoding phosphoenolpyruvate carboxylase and ATP citrate lyase, are missing. The involvement of the Wood–Ljungdahl pathway for CO_2_ fixation is unlikely, as no CO-methylating acetyl-CoA synthase was identified. Key rTCA cycle genes and CO-methylating acetyl-CoA synthase were not identified in *A. butzleri* hDNRA1, explaining its dependence on acetate for biomass carbon.

### Transcriptomic underpinning of hydrogenotrophic DNRA

Transcriptome sequencing yielded 73.2 ± 15.5 million reads per sample. Differential gene expression analysis revealed a substantial difference between the transcriptomes of *A. butzleri* hDNRA1 cultures grown with H_2_ (coupled with NO_3_^−^-to-NH_4_^+^ reduction) and those grown without H_2_ (NO_3_^−^-to-NO_2_^−^ reduction coupled with acetate oxidation) (Fig. 4B). Significant differences were observed for 1191 out of 2260 protein-coding genes, with 547 upregulated under the H_2_-oxidizing condition. The upregulation of the two sets of *hynAB* genes (3.7 ± 0.4/2.3 ± 0.4- and 6.2 ± 0.2/4.2 ± 0.2-fold; adjusted *P* value < .001) and the *nrfA* gene (4.0 ± 0.4-fold; adjusted *P* value < .001) suggested that the group 1b [NiFe]-hydrogenases and cytochrome *c*_552_ nitrite reductase catalyze H_2_ oxidation and NO_2_^−^-to-NH_4_^+^ reduction, respectively. The transcription of the *napAGHBFLD* cluster was constitutive and largely unaffected by H_2_ presence. The transcription of *napA* was at least 6.5-fold higher than those of the single-copy housekeeping genes *dnaA*, *rho*, and *recA* regardless of H_2_ presence, indicating that NO_3_^−^-to-NO_2_^−^ reduction is mediated by NapA. The downregulation of the *huaSL* genes (adjusted *P* value < .001) in the presence of H_2_ suggests that group 2d [NiFe] hydrogenase is unlikely to participate in hydrogenotrophic DNRA.

For *Sulfurospirillum* sp. hDNRA2, transcriptome data obtained from H_2_- and formate-fed cultures were compared, as formate was the only other electron donor that supported its anaerobic growth on NO_3_^−^ (Supplementary Fig. S4). Although the formate-amended cultures exhibited the phenotypes of DNRA *sensu stricto*, no significant difference was observed for *hynAB* transcription from the H_2_-grown cultures (adjusted *P* value > .05), suggesting that H_2_ produced from the formate hydrogen lyase complex could have been utilized as the electron donor for DNRA (Fig. 2D). Supporting this hypothesis, *fdhF* and *hycE*, encoding the catalytic subunits of formate dehydrogenase H and hydrogenase-3, respectively, were transcribed at a level comparable to those of the housekeeping gene *dnaA* (*P* value > .05), and up to 558 ± 108 μmol of H_2_ was detected during batch incubation with 5230 ± 90 μmol formate (Supplementary Fig. S2).

As formate condition was also considered as H_2_-dependent, transcriptome analysis focused on identifying genes that were highly expressed during hydrogenotrophic DNRA relative to single-copy housekeeping genes. Among the 2619 protein-coding genes, 426 (16.3%), including those encoding catalytic subunits of respiratory hydrogenases and DNRA-catalyzing enzymes, exhibited higher transcription levels than all scrutinized single-copy housekeeping genes (Supplementary Fig. S6). Both *hynAB* genes showed transcription levels at least 7.6-fold higher than that of *dnaA*. The *napA* and *nrfA* genes were among the top 4% of the most highly transcribed genes. One of the duplicate *nosZ* copies was also among the most highly expressed genes, explaining the transient N_2_O accumulation observed during hydrogenotrophic DNRA (Fig. 2F). In addition, genes encoding enzymes of the rTCA cycle, such as *sucCD* (succinate-CoA ligase), *gltA* (citrate synthase), *korAB* (2-oxoglutarate synthase), and *porA* (pyruvate synthase), all exhibited comparable or higher transcription levels than that of *recA* (Supplementary Fig. S7).

### Metagenomic surveys suggest ecological significance of hydrogenotrophic DNRA

**Figure 5:**
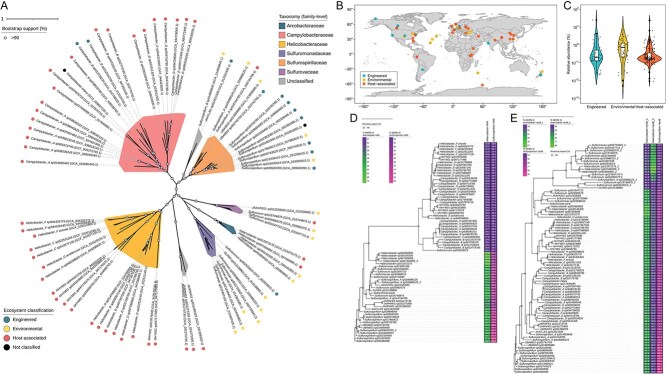
Analyses of Campylobacterota MAGs from the GTDB, containing at least one copy of each of the *napA*, *nrfA*, and *hynB* genes. (A) Maximum-likelihood phylogenetic tree of 74 Campylobacterota high-quality MAGs (CheckM completeness ≥ 90%, contamination ≤5%), constructed using IQ-TREE v2.3.6. Colored shades indicate family-level taxonomic affiliations, and colored dots next to the taxon descriptions represent the ecosystem classifications of their source metagenomes. (B) Geographical origins of the analyzed Campylobacterota MAGs, visualized on a world map. (C) Violin plots depicting the relative abundances (percentage of quality-trimmed raw reads from the source metagenome mapped onto each MAG) of Campylobacterota MAGs in their source metagenomes, categorized into three ecosystem types. Box plots within the violin plots display medians, interquartile ranges, and boundaries for non-outliers. (D, E) Maximum-likelihood phylogenetic trees constructed with *in-silico*-translated amino acid sequences of hydrogenotrophic DNRA marker genes *nrfA* (D) and *hynB* (E) identified from the Campylobacterota MAGs. Only non-truncated sequences are included. Heatmaps next to the trees indicate the amino acid identity between these sequences and those from *A*. *butzleri* hDNRA1 and *Sulfurospirillum* sp. hDNRA2.

Analyses of 475 Campylobacterota MAGs identified 75 MAGs harboring at least one copy of each of the *napA*, *nrfA*, and *hynB* genes (Fig. 5A). These MAGs were distributed across the phylum, covering six different previously defined families and three undefined family-level taxa, suggesting that the hydrogenotrophic DNRA genotype may be widespread among microorganisms within the Campylobacterota phylum.

The metagenomes from which these MAGs were derived include 142, 48, and 48 datasets from host-associated, environmental, and engineered ecosystems, respectively, categorized according to the JGI GOLD Ecosystem Classification criteria (accessed 8 November 2024; Fig. 5B and C; Supplementary Table S2). The putative hydrogenotrophic DNRA-catalyzing bacteria accounted for notable proportions of the microbial communities represented by these datasets, with 40 of 240 exhibiting >1% relative abundances. Several MAGs, found to be of substantial abundance in their respective metagenomes, were closely related to the two isolates examined in this study (Fig. 5D and E). For instance, a *Halarcobacter* MAG (GCA_002869565.1) harboring *nrfA* and *hynB* genes with >71% amino acid identity to those in *A. butzleri* hDNRA1 accounted for up to 4% of a laboratory enrichment of an estuary sediment. A *Sulfurospirillum* MAG (GCA_026988125.1), containing *nrfA* and *hynB* genes with >64% amino acid identity to those in *Sulfurospirillum* sp. hDNRA2, constituted 2% of the microbial population in a marine hydrothermal vent microbiome in the Mid-Atlantic Ridge.

A substantial proportion (41/75) of closely related MAGs affiliated with Campylobacteraceae and Helicobacteraceae originated from host-associated metagenomes. Nine of these MAGs, mostly recovered from mammalian digestive systems, had relative abundances of > 1%. The *nrfA* genes in these MAGs generally exhibited low similarity to those of the strains hDNRA1 and hDNRA2; however, they shared > 47% amino acid identity with the *nrfA* of a verified DNRA-catalyzing bacterium, *Campylobacter jejuni* NCTC11168 [55].

## Discussion

Thermodynamically and biochemically, the redox coupling of H_2_ oxidation and DNRA is difficult to be overlooked, as it is sufficiently exergonic, and various configurations of electron transport chains can bridge the substantial redox potential difference (Δ*E*_0_′ = 0.76 V) between the half-reactions [10, 56]. Several studies demonstrated bacterial isolates, including *Wolinella succinogenes*, capable of nitrate ammonification when supplied with H_2_ along with organic growth supplements, such as yeast extract [30–33, 57]. Other studies reported genomes and MAGs possessing both hydrogenase genes (e.g. *hynAB*) and *nrfA* [58, 59]. A recent study on the newly isolated *Trichlorobacter ammonificans*, possessing an octaheme cytochrome *c* nitrite reductase, suggested that H_2_ may serve as a supplemental electron donor for NO_2_^−^-to-NH_4_^+^ reduction [60]. Distinct from these previous studies, the current work demonstrates a reaction stoichiometry indicative of tight redox coupling between H_2_ oxidation and DNRA supporting cellular growth without the need for growth supplements containing costly biomass-building compounds or supplemental organic electron donors. Furthermore, these physiological findings are supported by genomic and transcriptomic data implicating group 1b [NiFe]-hydrogenases and NrfA in this pathway.

In the axenic cultures of the two isolates, hydrogenotrophic DNRA occurred independent of the presence of an organic e^−^ donor, seemingly challenging the widely accepted C:N ratio hypothesis on DNRA regulation [15, 19, 61]. Our findings suggest that hydrogenotrophic DNRA can be physiologically viable in environments with high H_2_ concentration but relatively low organic carbon availability, such as deep-sea hydrothermal vents [62]. Nevertheless, this does not necessarily invalidate the C:N ratio hypothesis, as potential H_2_ hotspots, often fueled by fermentative H_2_ production, overlap with environments characterized by high C:N ratios [63]. Furthermore, the C:N ratio hypothesis is theoretically based on the ecological competition between DNRA and denitrification to maximize energy acquisition from a limited resource, whether it is e^−^ donor (low C:N) or e^−^ acceptor (high C:N) [11, 13, 15]. Therefore, when an inorganic e^−^ donor is involved, the C:N ratio hypothesis should be considered in the context of e^−^ donor or e^−^ acceptor limitation, rather than the relative availability of organic carbon and nitrogenous e^−^ acceptors. How hydrogenotrophic DNRA and hydrogenotrophic denitrification compete for limiting H_2_ or NO_3_^−^ is yet unknown. Nonetheless, our observations from H_2_ oxidation biokinetics experiments and the NO_3_^−^-limiting chemostats suggest that the hydrogenotrophic DNRA pathway is a viable competitor under ecologically relevant H_2_-rich, NO_3_^−^-depleted environments.

Acetate was consumed alongside H_2_ in both isolates undergoing hydrogenotrophic DNRA, although the redox coupling of DNRA *sensu stricto* with H_2_ oxidation was clearly verified. Neither microorganism is, in that sense, autotrophic, although further examination with ^13^C-labeled substrates is warranted to determine whether they are mixotrophic or heterotrophic. The biomass yields observed for the two isolates examined here, based on the increase in OD_600_ value per 1 mM NO_3_^−^ reduced to NH_4_^+^, were approximately an order of magnitude lower than those observed in previously examined DNRA-catalyzing microorganisms belonging to the Pseudomonadota and Bacillota phyla, probably due to the energetic cost in incorporating acetate into biomass via acetyl-CoA synthesis [16, 17, 64]. However, as most, if not all, previous studies were performed with growth supplements, it would be premature to conclude that energy conservation in these hydrogenotrophic DNRA isolates is inherently less efficient than in organisms utilizing organoheterotrophic DNRA. Procurement of organic carbon sources by hydrogen-oxidizing bacteria has been previously observed [65, 66]. A previous study demonstrated, via quantitative stable isotope probing analysis, that addition of H_2_ increased the assimilation of ^13^C-acetate by certain bacterial taxa in microbiomes associated with deep-sea peridotite, which can serve as a source of H_2_ and acetate through serpentinization [67]. This H_2_ and acetate source may serve as the electron and carbon source, respectively, for *nrfA*-and-*hynAB*-possessing *Sulfurospirillum* spp. in the marine hydrothermal vent environments, where our metagenomic survey identified these organisms as abundant.

The two isolates, despite their genomic similarity (64.2% ANI) and sharing the same enzymes for H_2_ oxidation and DNRA, exhibited substantial physiological differences. They varied in their ability to use acetate as an electron donor for NO_3_^−^-to-NO_2_^−^ reduction and in their capacity to perform hydrogenotrophic DNRA without acetate. The extent of NO_2_^−^ accumulation during NO_3_^−^-to-NH_4_^+^ reduction also differed, suggesting that hydrogenotrophic DNRA in *Sulfurillospirillum* sp. hDNRA2 was not inhibited by NO_3_^−^, unlike *A. butzleri* hDNRA1 or previously studied organotrophic DNRA-catalyzing bacteria [16, 68]. These subtle but significant physiological differences cannot be predicted by genomic analysis alone, highlighting the importance of cultivation in advancing our understanding of biogeochemical processes and unlocking the biotechnological potential of microorganisms and microbiota in nature [69].

The metagenomic survey revealed that genomic features of hydrogenotrophic DNRA are widespread among microorganisms affiliated with Campylobacterota. Several Campylobacterota MAGs harboring *napA*, *nrfA*, and *hynB*, including several closely associated with *Sulfurospirillum* sp. hDNRA2, had been derived from metagenomes of deep-sea hydrothermal vent microbiomes, where they constituted a significant subpopulation [70, 71]. Both the *nrfA* and *hynAB* genes of the *Sulfurospirillum* MAGs exhibited high similarity to those of *Sulfurospillum* sp. hDNRA2, suggesting a comparable metabolic capability for hydrogenotrophic DNRA. Hydrothermal vents are well-known hotspots for H_2_-oxiding microorganisms utilizing H_2_ carried by hydrothermal fluids, and NO_3_^−^ is readily available in seawater [72, 73]. That NH_4_^+^ is generated biologically from seawater NO_3_^−^ in such environment was previously evidenced from isotopic analyses of NH_4_^+^ sampled from hydrothermal vents on the Juan de Fuca Ridge [74]. Furthermore, several thermophilic bacterial isolates from deep-sea hydrothermal vents have demonstrated the capability to couple H_2_ oxidation with nitrate ammonification, despite lacking known ammonia-forming nitrite reductases in their genomes [30, 33, 57]. Although the *K*_*m*(app)_ value of *Sulfurospirillum* sp. hDNRA2 did not indicate a high affinity to H_2_, micromolar concentration of H_2_, sufficiently high to support its growth, is not anomalous in vicinity of hydrothermal vents [67, 75]. Thus, hydrogenotrophic DNRA by *Sulfurospirillum* spp. may be among contributors to NH_4_^+^ generation in the hydrothermal vent environments.

The MAGs closely affiliated with *Sulfurospirillum* sp. hDNRA2 were also abundant in highly reduced, organic- rich samples from engineered environments, such as landfills and oil production facilities. In addition, several MAGs affiliated to *Helicobacter* and *Campylobacter* were identified with high relative abundance in the metagenomes derived from mammalian digestive systems. Alteration of nitrogen cycling would probably not be ecologically significant in these environments; however, hydrogenotrophic DNRA metabolism may play other consequential roles, such as competing for H_2_ with anaerobic biogeochemical reactions of ecological and environmental importance like hydrogenotrophic methanogenesis, or aiding the survival of opportunistic pathogens in mammalian hosts [76, 77]. In addition, these environments could serve as a promising starting point for discovering new hydrogenotrophic DNRA-catalyzing isolates with fascinating physiological diversities. All these possibilities offer compelling opportunities for future research that would enhance our understanding of nitrogen- and hydrogen-cycling in diverse ecosystems.

## Supplementary Material

Supplementary_Material_wraf092_(revised)

## Data Availability

The complete genomes and transcriptomic raw sequencing datasets generated during and/or analyzed during the current study were deposited in NCBI’s GenBank and Sequence Read Archive (SRA) databases, respectively (Accessions: CP175552.1 and CP159800.1 for complete genomes, SRX25285529/SRX25285530 and SRX25286366/SRX25286367 for transcriptomes).
