# Supplementary material for: Hydrogen-dependent dissimilatory nitrate reduction to ammonium enables growth of Campylobacterota isolates
Source: ISME J. 2025 May 14;19(1):wraf092. doi: 10.1093/ismejo/wraf092 (PMC12286921; doi:10.1093/ismejo/wraf092)
Supplement: Supplementary_Material_wraf092_(revised) [file supplementary_material_wraf092_(revised).docx]

**Supplementary Material**

**Hydrogen-dependent dissimilatory nitrate reduction to ammonium enables growth of Campylobacterota isolates**

Hokwan Heo^1^, Thanh Nguyen-Dinh^2^, Man-Young Jung^3,4^, Chris Greening^2^, and Sukhwan Yoon^1,#^

^1^Department of Civil and Environmental Engineering, Korea Advanced Institute of Science and Technology (KAIST), Daejeon 34141, Republic of Korea

^2^Department of Microbiology, Biomedicine Discovery Institute, Monash University, Clayton, VIC 3800, Australia.

^3^Interdisciplinary Graduate Program in Advance Convergence Technology and Science, Jeju National University, Jeju 63243, Republic of Korea

^4^Department of Biology Education, Jeju National University, Jeju 63243, Republic of Korea

^#^Corresponding author:

Sukhwan Yoon (E-mail: syoon80@kaist.ac.kr)

Department of Civil and Environmental Engineering

Korea Advanced Institute of Science and Technology (KAIST)

291, Daehak-ro, Yuseong-gu, Daejeon 34141, Republic of Korea

**Supplementary figures**


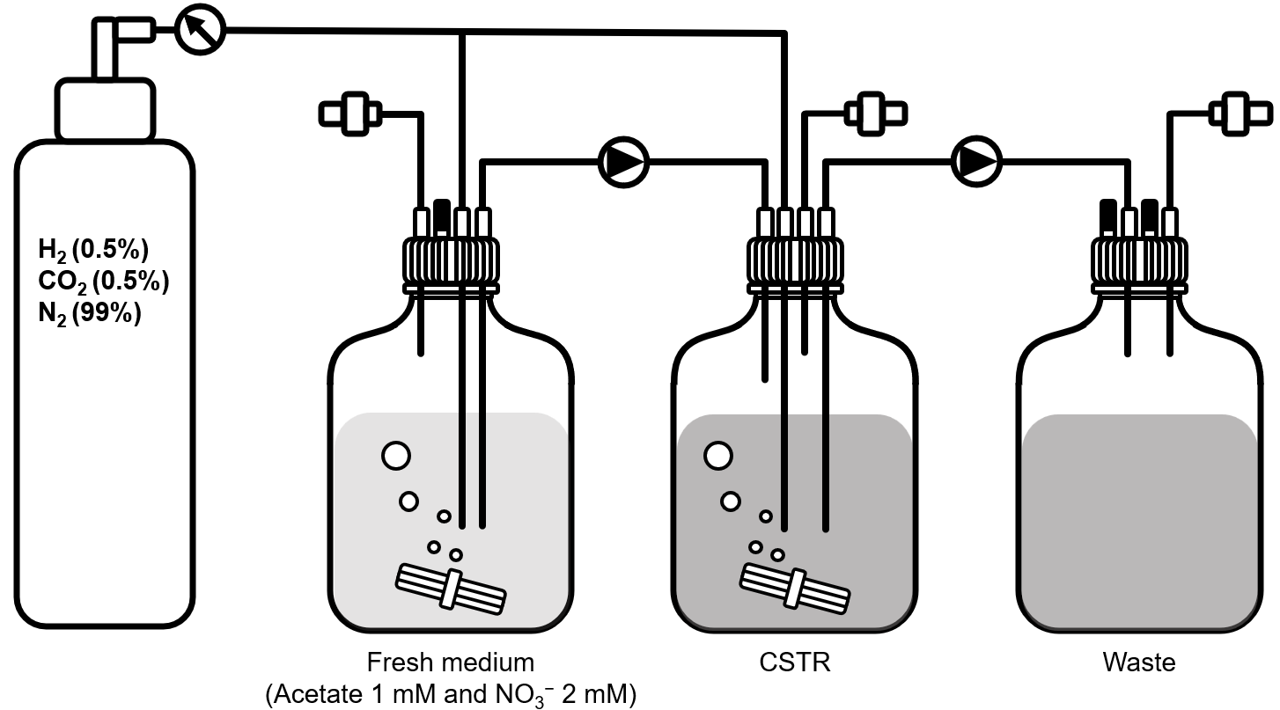


Fig. S1. Schematic depiction of the chemostat reactor used for the continuous cultivation of *A*. *butzleri* hDNRA1 and *Sulfurospirillum* sp. hDNRA2 under hydrogenotrophic DNRA conditions.


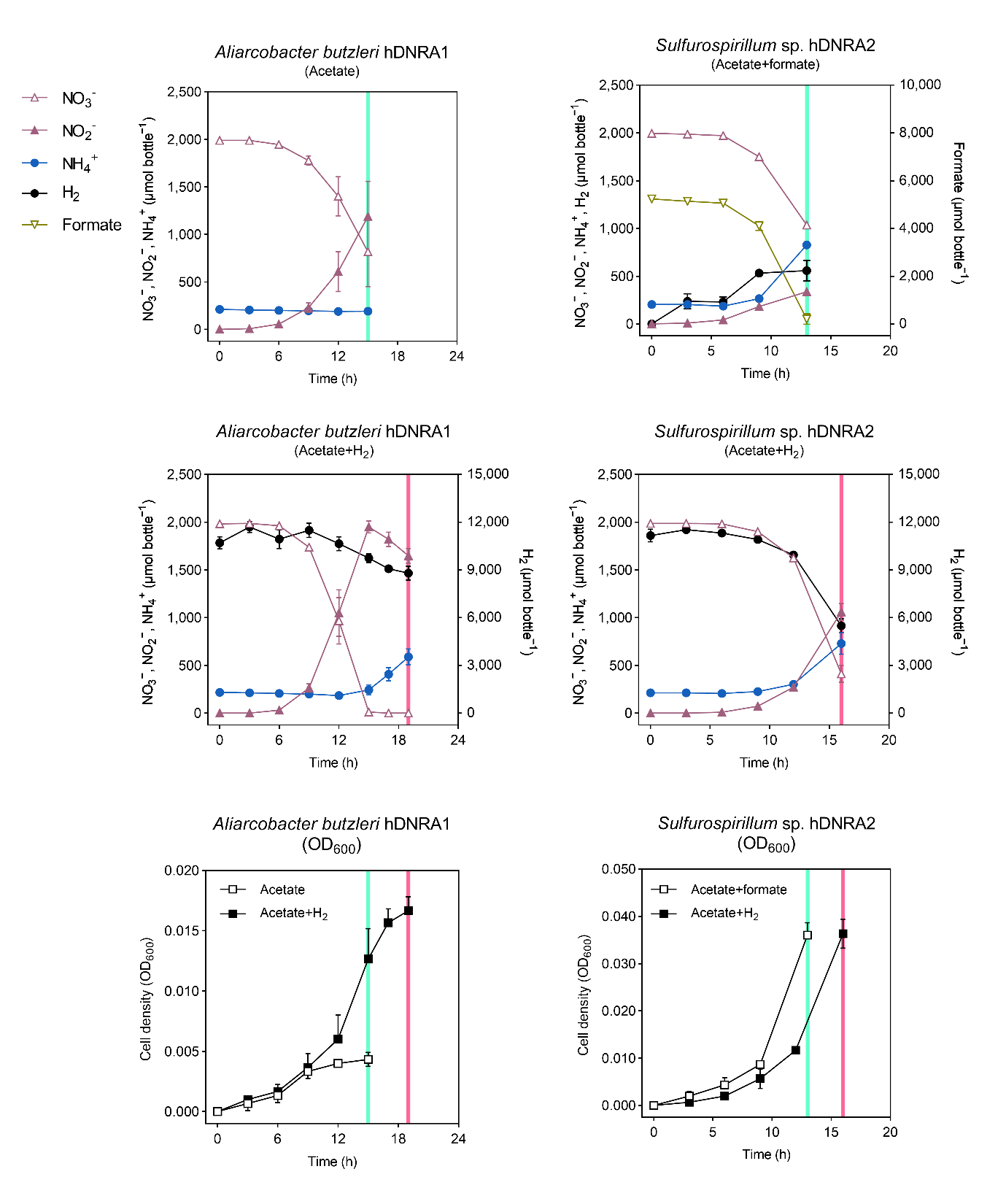


Fig. S2. Culture incubation and sample collection for transcriptome analysis. Batch cultures of *A*. *butzleri* (left column) hDNRA1 and *Sulfurospirillum* sp. hDNRA2 (right column) were incubated in medium amended with 1 mM acetate and 2 mM NO_3_^−^. For the H₂-free *Sulfurospirillum* sp. hDNRA2 control, 5 mM formate was added, and cultures were equilibrated with either 5% H₂/5% CO₂/90% N₂ or 5% CO₂/95% N₂ (controls without H₂) mixed gas. The concentrations of NO_3_^−^, NO_2_^−^, NH_4_^+^, H_2_, formate, and cell density, were monitored until (i) active NH_4_^+^ production, (ii) NO_3_^−^ reduction, and/or (iii) exponential growth was observed. Vertical lines indicate the time points for RNA sampling. Each data point represents the mean of three biological replicates (*n*=3), with samples separately collected, treated, and sequenced, and error bars indicating standard deviations.


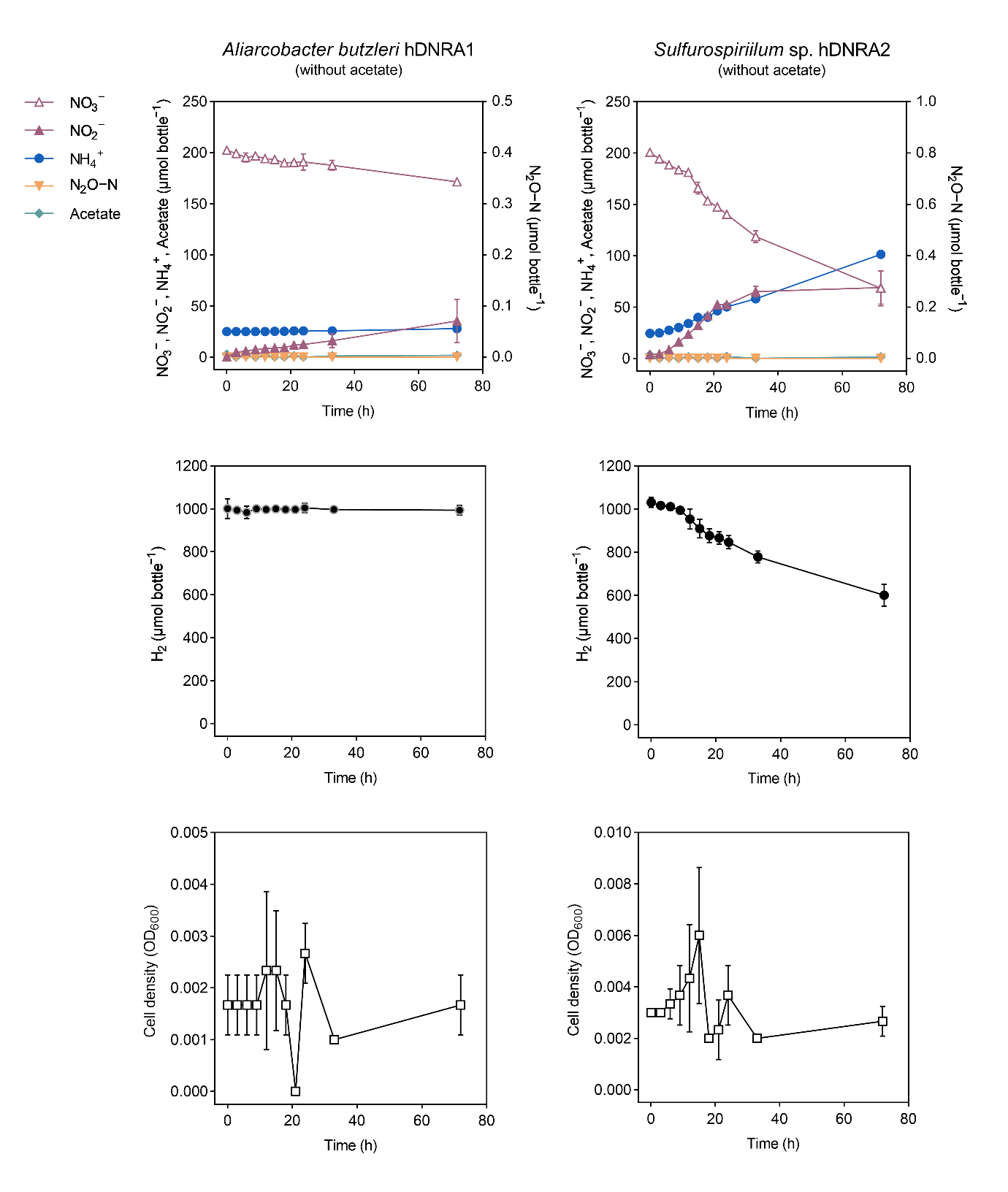


Fig. S3. Hydrogenotrophic DNRA activity in batch cultures of *A*. *butzleri* hDNRA1 (left column) and *Sulfurospirillum* sp. hDNRA2 (right column) incubated in the absence of organic carbon. Cultures were prepared with washed cells resuspended in fresh medium initially containing 2 mM NO_3_^−^ but lacking acetate, with an initial cell density of OD_600_ ~0.002. The initial headspace contained 5% H_2_ and 5% CO_2_ (v/v). The concentrations of NO_3_^−^, NO_2_^−^, NH_4_^+^, N_2_O-N, H_2_ in the bottle, and cell density, were monitored over a 72-hour period. Each data point represents the mean of three biological replicates (*n*=3), with error bars indicating standard deviations.


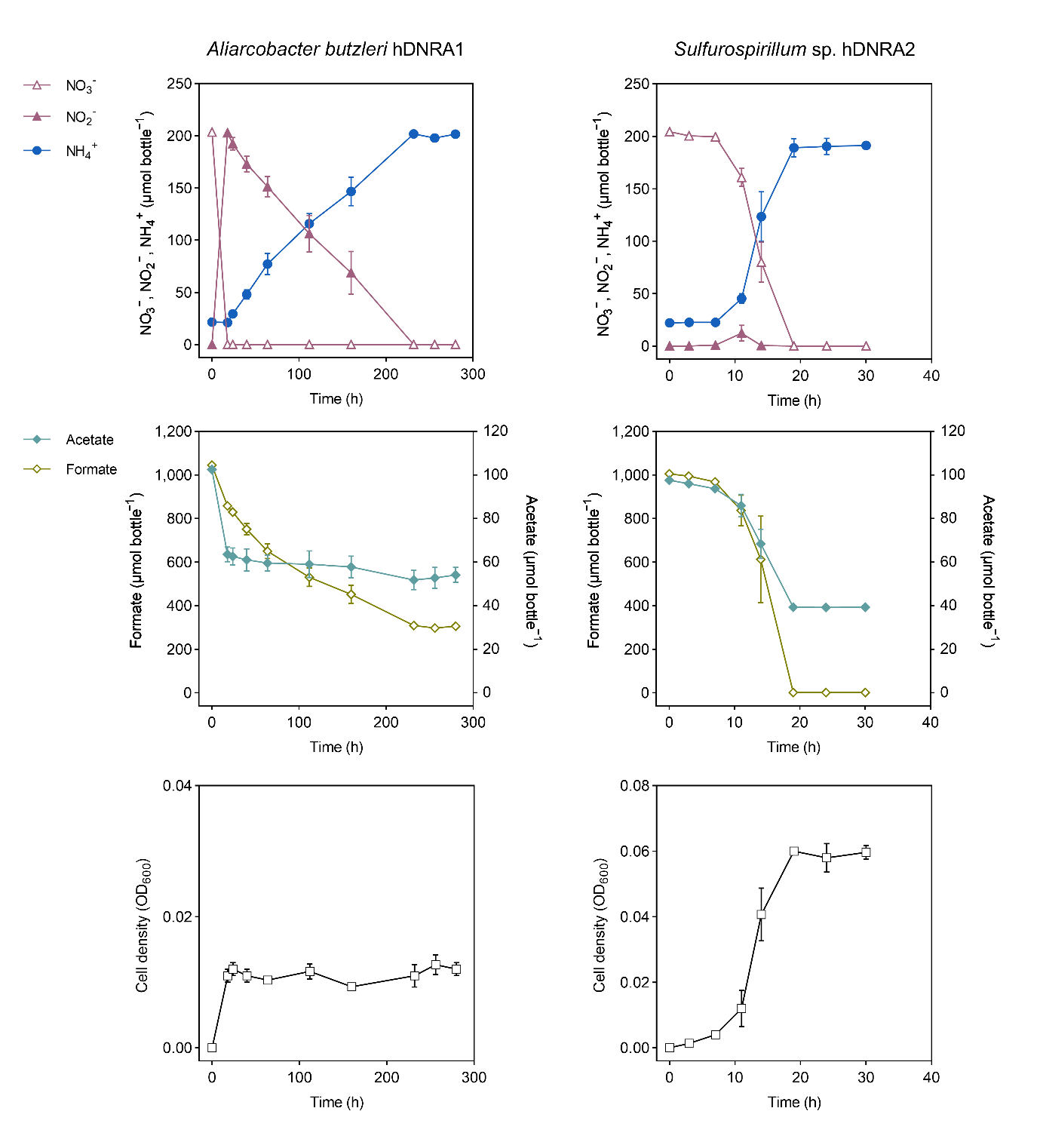


Fig. S4. Growth and NO_3_^−^/NO_2_^−^ reduction by *A*. *butzleri* hDNRA1 (left column) and *Sulfurospirillum* sp. hDNRA2 (right column) observed in batch cultures initially amended with 2 mM NO_3_^−^, 10 mM formate, and 1 mM acetate, but without H_2_ in the headspace. Cultures equilibrated with CO_2_/N_2_ (5:95, v/v) were examined. The concentrations of NO_3_^−^, NO_2_^−^, NH_4_^+^, acetate, formate, and cell density (measured as OD_600_) were monitored until NO_3_^−^ and NO_2_^−^ were depleted. Each data point represents the mean of three biological replicates (*n*=3), with error bars indicating standard deviations.


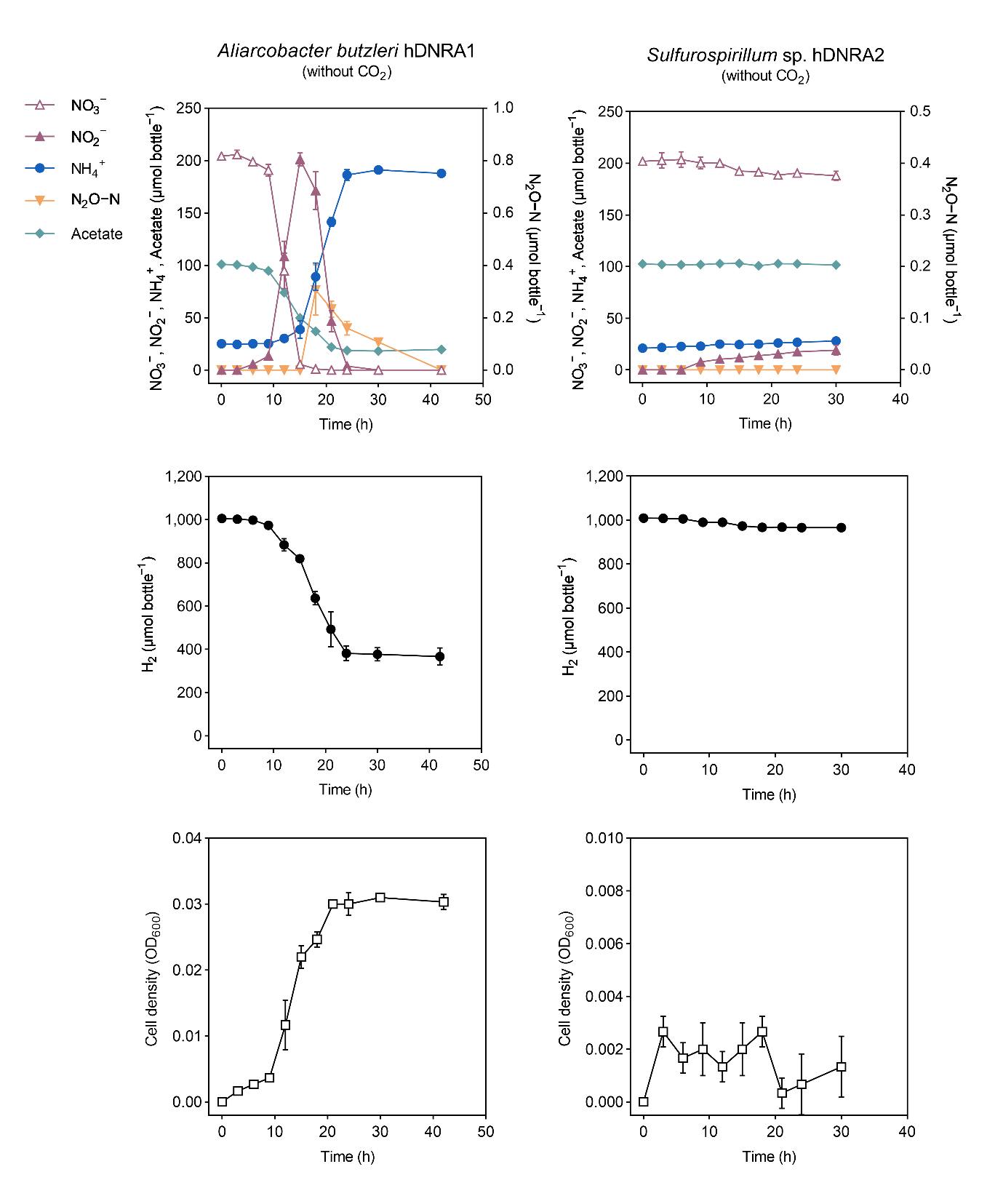


Fig. S5. Hydrogenotrophic DNRA activity in batch cultures of *A*. *butzleri* hDNRA1 (left column) and *Sulfurospirillum* sp. hDNRA2 (right column) incubated under initial absence of inorganic carbon. Transformation of NO_3_^−^ and associated H_2_ consumption was examined in batch cultures prepared with 2 mM NO_3_^−^, 1 mM acetate, and a CO_2_-free headspace (5% H_2_/ 95% N_2_, v/v). The concentrations of NO_3_^−^, NO_2_^−^, NH_4_^+^, N_2_O-N, H_2_ in the bottle, and cell density, were monitored until the depletion of NO_3_^−^ and NO_2_^−^. Each data point represents the mean of three biological replicates (*n*=3), with error bars indicating standard deviations.


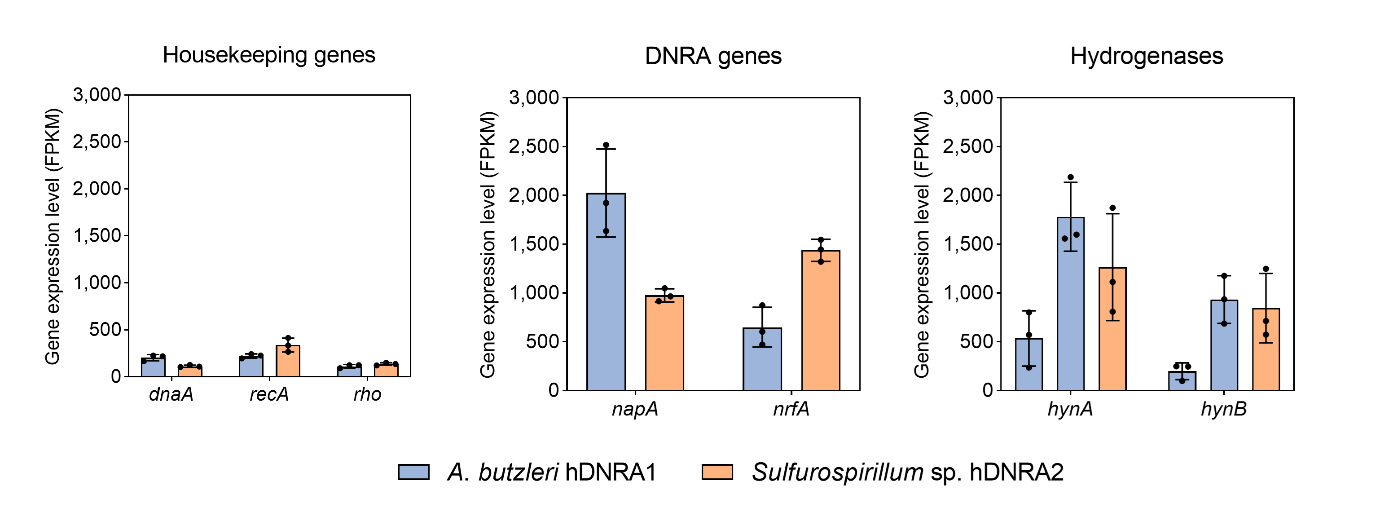


Fig. S6. Expression levels of the genes putatively involved in DNRA and H_2_ oxidation compared with those of three single-copy housekeeping genes *dnaA*, *recA*, and *rho*, as computed from the sequenced transcriptomes of *A*. *butzleri* hDNRA1 and *Sulfurospirillum* sp. hDNRA2 grown on hydrogenotrophic DNRA. The expression levels of the genes encoding the periplasmic nitrate reductase (*napA*), cytochrome *c*_552_ nitrite reductase (*nrfA*), and group 2d [NiFe]-hydrogenase (*hynAB*) are presented, along with those of the three single-copy housekeeping genes. Note that the complete genome of *A*. *butzleri* hDNRA1 contains two sets of *hynAB* genes, while all other genes examined here are single-copy in their respective genomes. Each bar length represents the mean of three biological replicates (*n*=3; each shown as a black dot) with error bars indicating standard deviations.


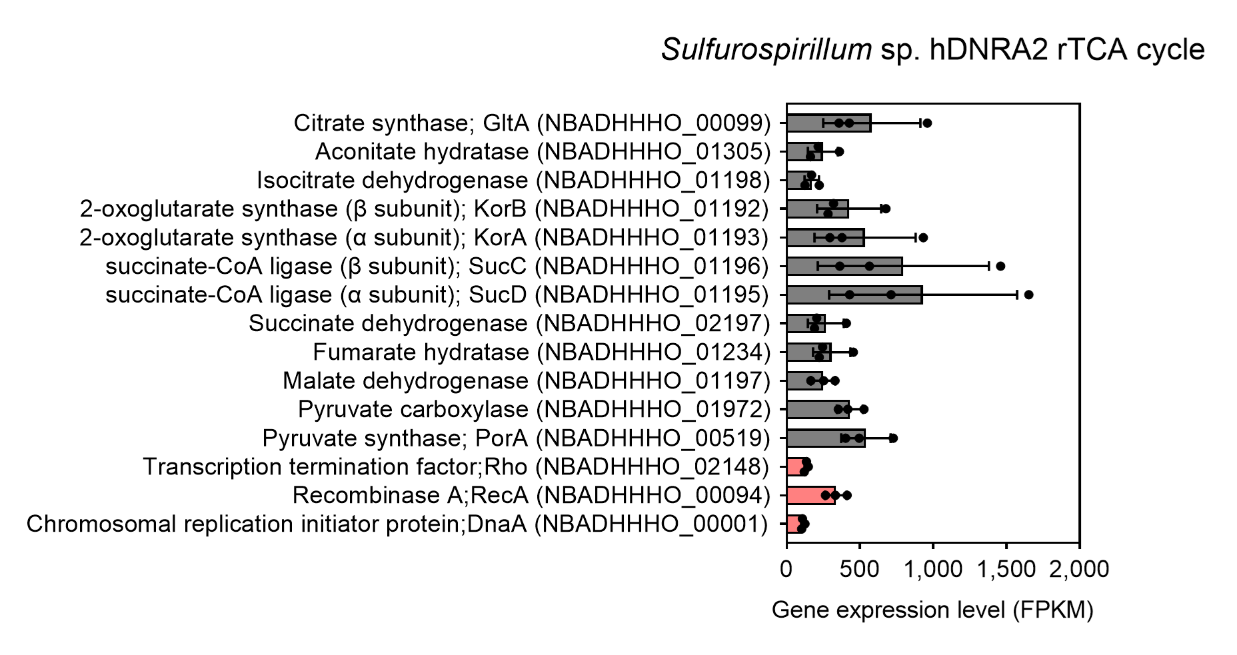


Fig. S7. Expression levels of the genes putatively involved in the reductive TCA (rTCA) pathway in transcriptome of *Sulfurospirillum* sp. hDNRA2 grown under hydrogenotrophic DNRA growth condition. Expression levels of single-copy housekeeping genes, *dnaA*, *recA*, and *rho* are also shown for comparison (red bar plots). Each bar length represents the mean of three biological replicates (*n*=3; each shown as a black dot) with error bars indicating standard deviations.

**Supplementary tables**

Table S1. Composition of 1000× trace element solution.

| Chemical | Amounts (g per litre of deionized water) |
| --- | --- |
| MgSO_4_ · 7H_2_O | 30.0 |
| MnSO_4_ · H_2_O | 5.0 |
| NaCl | 10.0 |
| FeSO_4_ · 7H_2_O | 1.0 |
| CaCl_2_ · 2H_2_O | 1.0 |
| CoCl_2_ · 6H_2_O | 1.0 |
| ZnCl_2_ | 1.0 |
| CuSO_4_ · 5H_2_O | 0.1 |
| AlK(SO_4_)_2_ · 12H_2_O | 0.1 |
| H_3_BO_3_ | 0.1 |
| Na_2_MoO_4_ · 2H_2_O | 0.25 |
| NiCl_2_ · 6H_2_O | 0.24 |
| Na_2_WO_4_ · 2H_2_O | 0.25 |
| Na_2_SeO_3_ | 0.18 |

Table S2. Metagenomic origin, relative abundance, and ecosystem classification of 75 *Campylobacteroata* MAGs used in this study.

| **#** | **Accession** | **GTDB Taxonomy** | **Latitude (°)** | **Longitude (°)** | **Isolation source** | **Ecosystem type** | **% mapped reads** |
| --- | --- | --- | --- | --- | --- | --- | --- |
| 1 | GCA_001602955.1 | d__Bacteria; p__Campylobacterota; c__Campylobacteria; o__Campylobacterales; f__Sulfurospirillaceae; g__Sulfurospirillum; s__Sulfurospirillum sp001602955 | N/A | N/A | anaerobic digester (cellulose-degrading) | N/A | - |
| 2 | GCA_002328495.1 | d__Bacteria; p__Campylobacterota; c__Campylobacteria; o__Campylobacterales; f__Sulfurospirillaceae; g__Sulfurospirillum; s__Sulfurospirillum sp002328495 | 57.020000 | -111.550000 | suncor tailing pond 6 meters | Engineered;Built environment;Tailings pond;Wastewater;Unclassified | 0.124% |
|  | GCA_002328495.1 | d__Bacteria; p__Campylobacterota; c__Campylobacteria; o__Campylobacterales; f__Sulfurospirillaceae; g__Sulfurospirillum; s__Sulfurospirillum sp002328495 | 57.020000 | -111.550000 | suncor tailing pond 6 meters | Engineered;Built environment;Tailings pond;Wastewater;Unclassified | 0.124% |
|  | GCA_002328495.1 | d__Bacteria; p__Campylobacterota; c__Campylobacteria; o__Campylobacterales; f__Sulfurospirillaceae; g__Sulfurospirillum; s__Sulfurospirillum sp002328495 | 57.020000 | -111.550000 | suncor tailing pond 6 meters | Engineered;Built environment;Tailings pond;Wastewater;Unclassified | 0.124% |
|  | GCA_002328495.1 | d__Bacteria; p__Campylobacterota; c__Campylobacteria; o__Campylobacterales; f__Sulfurospirillaceae; g__Sulfurospirillum; s__Sulfurospirillum sp002328495 | 57.020000 | -111.550000 | suncor tailing pond 6 meters | Engineered;Built environment;Tailings pond;Wastewater;Unclassified | 0.126% |
|  | GCA_002328495.1 | d__Bacteria; p__Campylobacterota; c__Campylobacteria; o__Campylobacterales; f__Sulfurospirillaceae; g__Sulfurospirillum; s__Sulfurospirillum sp002328495 | 57.020000 | -111.550000 | suncor tailing pond 6 meters | Engineered;Built environment;Tailings pond;Wastewater;Unclassified | 0.125% |
|  | GCA_002328495.1 | d__Bacteria; p__Campylobacterota; c__Campylobacteria; o__Campylobacterales; f__Sulfurospirillaceae; g__Sulfurospirillum; s__Sulfurospirillum sp002328495 | 57.020000 | -111.550000 | suncor tailing pond 6 meters | Engineered;Built environment;Tailings pond;Wastewater;Unclassified | 0.124% |
|  | GCA_002328495.1 | d__Bacteria; p__Campylobacterota; c__Campylobacteria; o__Campylobacterales; f__Sulfurospirillaceae; g__Sulfurospirillum; s__Sulfurospirillum sp002328495 | 57.020000 | -111.550000 | suncor tailing pond 6 meters | Engineered;Built environment;Tailings pond;Wastewater;Unclassified | 0.125% |
| 3 | GCA_002382085.1 | d__Bacteria; p__Campylobacterota; c__Campylobacteria; o__Campylobacterales; f__Sulfurospirillaceae; g__Sulfurospirillum; s__Sulfurospirillum sp002382085 | 57.020000 | -111.550000 | suncor tailing pond 6 meters | Engineered;Built environment;Tailings pond;Wastewater;Unclassified | 0.751% |
| 4 | GCA_002733945.2 | d__Bacteria; p__Campylobacterota; c__Campylobacteria; o__Campylobacterales; f__Sulfurimonadaceae; g__Sulfurimonas; s__Sulfurimonas sp002733945 | 22.800000 | -46.050000 | marine subsurface aquifer | Environmental;Aquatic;Marine;Deep subsurface;Unclassified | 1.125% |
|  | GCA_002733945.2 | d__Bacteria; p__Campylobacterota; c__Campylobacteria; o__Campylobacterales; f__Sulfurimonadaceae; g__Sulfurimonas; s__Sulfurimonas sp002733945 | 22.800000 | -46.050000 | marine subsurface aquifer | Environmental;Aquatic;Marine;Deep subsurface;Unclassified | 1.300% |
|  | GCA_002733945.2 | d__Bacteria; p__Campylobacterota; c__Campylobacteria; o__Campylobacterales; f__Sulfurimonadaceae; g__Sulfurimonas; s__Sulfurimonas sp002733945 | 22.780000 | -46.090000 | marine subsurface aquifer | Environmental;Aquatic;Marine;Deep subsurface;Unclassified | 0.551% |
|  | GCA_002733945.2 | d__Bacteria; p__Campylobacterota; c__Campylobacteria; o__Campylobacterales; f__Sulfurimonadaceae; g__Sulfurimonas; s__Sulfurimonas sp002733945 | 22.780000 | -46.090000 | marine subsurface aquifer | Environmental;Aquatic;Marine;Deep subsurface;Unclassified | 0.725% |
|  | GCA_002733945.2 | d__Bacteria; p__Campylobacterota; c__Campylobacteria; o__Campylobacterales; f__Sulfurimonadaceae; g__Sulfurimonas; s__Sulfurimonas sp002733945 | 22.780000 | -46.090000 | marine subsurface aquifer | Environmental;Aquatic;Marine;Deep subsurface;Unclassified | 0.152% |
|  | GCA_002733945.2 | d__Bacteria; p__Campylobacterota; c__Campylobacteria; o__Campylobacterales; f__Sulfurimonadaceae; g__Sulfurimonas; s__Sulfurimonas sp002733945 | 22.780000 | -46.090000 | marine subsurface aquifer | Environmental;Aquatic;Marine;Deep subsurface;Unclassified | 0.312% |
|  | GCA_002733945.2 | d__Bacteria; p__Campylobacterota; c__Campylobacteria; o__Campylobacterales; f__Sulfurimonadaceae; g__Sulfurimonas; s__Sulfurimonas sp002733945 | 22.780000 | -46.090000 | marine subsurface aquifer | Environmental;Aquatic;Marine;Deep subsurface;Unclassified | 0.386% |
|  | GCA_002733945.2 | d__Bacteria; p__Campylobacterota; c__Campylobacteria; o__Campylobacterales; f__Sulfurimonadaceae; g__Sulfurimonas; s__Sulfurimonas sp002733945 | 22.780000 | -46.090000 | marine subsurface aquifer | Environmental;Aquatic;Marine;Deep subsurface;Unclassified | 0.325% |
|  | GCA_002733945.2 | d__Bacteria; p__Campylobacterota; c__Campylobacteria; o__Campylobacterales; f__Sulfurimonadaceae; g__Sulfurimonas; s__Sulfurimonas sp002733945 | 22.780000 | -46.090000 | marine subsurface aquifer | Environmental;Aquatic;Marine;Deep subsurface;Unclassified | 1.108% |
|  | GCA_002733945.2 | d__Bacteria; p__Campylobacterota; c__Campylobacteria; o__Campylobacterales; f__Sulfurimonadaceae; g__Sulfurimonas; s__Sulfurimonas sp002733945 | 22.780000 | -46.090000 | marine subsurface aquifer | Environmental;Aquatic;Marine;Deep subsurface;Unclassified | 0.291% |
|  | GCA_002733945.2 | d__Bacteria; p__Campylobacterota; c__Campylobacteria; o__Campylobacterales; f__Sulfurimonadaceae; g__Sulfurimonas; s__Sulfurimonas sp002733945 | 22.800000 | -46.050000 | marine subsurface aquifer | Environmental;Aquatic;Marine;Deep subsurface;Unclassified | 0.810% |
|  | GCA_002733945.2 | d__Bacteria; p__Campylobacterota; c__Campylobacteria; o__Campylobacterales; f__Sulfurimonadaceae; g__Sulfurimonas; s__Sulfurimonas sp002733945 | 22.800000 | -46.050000 | marine subsurface aquifer | Environmental;Aquatic;Marine;Deep subsurface;Unclassified | 0.393% |
|  | GCA_002733945.2 | d__Bacteria; p__Campylobacterota; c__Campylobacteria; o__Campylobacterales; f__Sulfurimonadaceae; g__Sulfurimonas; s__Sulfurimonas sp002733945 | 22.780000 | -46.090000 | marine subsurface aquifer | Environmental;Aquatic;Marine;Deep subsurface;Unclassified | 0.019% |
|  | GCA_002733945.2 | d__Bacteria; p__Campylobacterota; c__Campylobacteria; o__Campylobacterales; f__Sulfurimonadaceae; g__Sulfurimonas; s__Sulfurimonas sp002733945 | 22.800000 | -46.050000 | marine subsurface aquifer | Environmental;Aquatic;Marine;Deep subsurface;Unclassified | 2.953% |
|  | GCA_002733945.2 | d__Bacteria; p__Campylobacterota; c__Campylobacteria; o__Campylobacterales; f__Sulfurimonadaceae; g__Sulfurimonas; s__Sulfurimonas sp002733945 | 22.800000 | -46.050000 | marine subsurface aquifer | Environmental;Aquatic;Marine;Deep subsurface;Unclassified | 0.204% |
|  | GCA_002733945.2 | d__Bacteria; p__Campylobacterota; c__Campylobacteria; o__Campylobacterales; f__Sulfurimonadaceae; g__Sulfurimonas; s__Sulfurimonas sp002733945 | 22.800000 | -46.050000 | marine subsurface aquifer | Environmental;Aquatic;Marine;Deep subsurface;Unclassified | 0.246% |
|  | GCA_002733945.2 | d__Bacteria; p__Campylobacterota; c__Campylobacteria; o__Campylobacterales; f__Sulfurimonadaceae; g__Sulfurimonas; s__Sulfurimonas sp002733945 | 22.780000 | -46.090000 | marine subsurface aquifer | Environmental;Aquatic;Marine;Deep subsurface;Unclassified | 0.250% |
| 5 | GCA_002742765.1 | d__Bacteria; p__Campylobacterota; c__Campylobacteria; o__Campylobacterales; f__Sulfurospirillaceae; g__UBA6810; s__UBA6810 sp002742765 | 71.170000 | -156.450000 | oil production facility | Engineered;Built environment;Oil refinery;Unclassified;Unclassified | 2.809% |
|  | GCA_002742765.1 | d__Bacteria; p__Campylobacterota; c__Campylobacteria; o__Campylobacterales; f__Sulfurospirillaceae; g__UBA6810; s__UBA6810 sp002742765 | 71.170000 | -156.450000 | oil production facility | Engineered;Built environment;Oil refinery;Unclassified;Unclassified | 3.398% |
|  | GCA_002742765.1 | d__Bacteria; p__Campylobacterota; c__Campylobacteria; o__Campylobacterales; f__Sulfurospirillaceae; g__UBA6810; s__UBA6810 sp002742765 | 71.170000 | -156.450000 | oil production facility | Engineered;Built environment;Oil refinery;Unclassified;Unclassified | 0.501% |
| 6 | GCA_002869565.1 | d__Bacteria; p__Campylobacterota; c__Campylobacteria; o__Campylobacterales; f__Arcobacteraceae; g__Halarcobacter; s__Halarcobacter sp002869565 | 37.862900 | -122.313200 | enrichment from estuary sediment | Engineered;Lab enrichment;Unclassified;Unclassified;Unclassified | 4.264% |
|  | GCA_002869565.1 | d__Bacteria; p__Campylobacterota; c__Campylobacteria; o__Campylobacterales; f__Arcobacteraceae; g__Halarcobacter; s__Halarcobacter sp002869565 | 37.862900 | -122.313200 | enrichment from estuary sediment | Engineered;Lab enrichment;Unclassified;Unclassified;Unclassified | 0.137% |
|  | GCA_002869565.1 | d__Bacteria; p__Campylobacterota; c__Campylobacteria; o__Campylobacterales; f__Arcobacteraceae; g__Halarcobacter; s__Halarcobacter sp002869565 | 37.862900 | -122.313200 | enrichment from estuary sediment | Engineered;Lab enrichment;Unclassified;Unclassified;Unclassified | 0.947% |
|  | GCA_002869565.1 | d__Bacteria; p__Campylobacterota; c__Campylobacteria; o__Campylobacterales; f__Arcobacteraceae; g__Halarcobacter; s__Halarcobacter sp002869565 | 37.862900 | -122.313200 | enrichment from estuary sediment | Engineered;Lab enrichment;Unclassified;Unclassified;Unclassified | 0.726% |
|  | GCA_002869565.1 | d__Bacteria; p__Campylobacterota; c__Campylobacteria; o__Campylobacterales; f__Arcobacteraceae; g__Halarcobacter; s__Halarcobacter sp002869565 | 37.862900 | -122.313200 | enrichment from estuary sediment | Environmental;Aquatic;Marine;Intertidal zone;Estuary: Sediment | 0.035% |
| 7 | GCA_009827235.1 | d__Bacteria; p__Campylobacterota; c__Campylobacteria; o__Campylobacterales; f__Campylobacteraceae; g__Campylobacter; s__Campylobacter infans | N/A | N/A | infant under 1 year of age, breastfeeding, had prelonged diarrhea | N/A | - |
| 8 | GCA_009930355.1 | d__Bacteria; p__Campylobacterota; c__Campylobacteria; o__Campylobacterales; f__Sulfurospirillaceae; g__Sulfurospirillum; s__Sulfurospirillum sp009930355 | -31.201857 | -61.822226 | first pond of the AUR serial dairy industry stabilization pond system | Engineered;Wastewater;Industrial wastewater;Tailings pond;Unclassified | 2.150% |
| 9 | GCA_012329065.1 | d__Bacteria; p__Campylobacterota; c__Campylobacteria; o__Campylobacterales; f__Sulfurovaceae; g__JAAXPZ01; s__JAAXPZ01 sp012329065 | 30.124051 | -42.120113 | marine hydrothermal vent chimney | Environmental;Aquatic;Marine;Hydrothermal vents;Unclassified | 0.733% |
| 10 | GCA_012519705.1 | d__Bacteria; p__Campylobacterota; c__Campylobacteria; o__Campylobacterales; f__Campylobacteraceae; g__Campylobacter_B; s__Campylobacter_B sp012519705 | 52.038500 | 8.495600 | anaerobic digestion of organic wastes under variable temperature conditions and feedstocks | Engineered;Bioreactor;Anaerobic;Digestate;Unclassified | 0.312% |
|  | GCA_012519705.1 | d__Bacteria; p__Campylobacterota; c__Campylobacteria; o__Campylobacterales; f__Campylobacteraceae; g__Campylobacter_B; s__Campylobacter_B sp012519705 | 52.038500 | 8.495600 | anaerobic digestion of organic wastes under variable temperature conditions and feedstocks | Engineered;Bioreactor;Anaerobic;Digestate;Unclassified | 0.445% |
|  | GCA_012519705.1 | d__Bacteria; p__Campylobacterota; c__Campylobacteria; o__Campylobacterales; f__Campylobacteraceae; g__Campylobacter_B; s__Campylobacter_B sp012519705 | 52.038500 | 8.495600 | anaerobic digestion of organic wastes under variable temperature conditions and feedstocks | Engineered;Bioreactor;Anaerobic;Digestate;Unclassified | 0.026% |
|  | GCA_012519705.1 | d__Bacteria; p__Campylobacterota; c__Campylobacteria; o__Campylobacterales; f__Campylobacteraceae; g__Campylobacter_B; s__Campylobacter_B sp012519705 | 52.038500 | 8.495600 | anaerobic digestion of organic wastes under variable temperature conditions and feedstocks | Engineered;Bioreactor;Anaerobic;Digestate;Unclassified | 0.027% |
|  | GCA_012519705.1 | d__Bacteria; p__Campylobacterota; c__Campylobacteria; o__Campylobacterales; f__Campylobacteraceae; g__Campylobacter_B; s__Campylobacter_B sp012519705 | 52.038500 | 8.495600 | anaerobic digestion of organic wastes under variable temperature conditions and feedstocks | Engineered;Bioreactor;Anaerobic;Digestate;Unclassified | 0.507% |
|  | GCA_012519705.1 | d__Bacteria; p__Campylobacterota; c__Campylobacteria; o__Campylobacterales; f__Campylobacteraceae; g__Campylobacter_B; s__Campylobacter_B sp012519705 | 52.038500 | 8.495600 | anaerobic digestion of organic wastes under variable temperature conditions and feedstocks | Engineered;Bioreactor;Anaerobic;Digestate;Unclassified | 0.368% |
|  | GCA_012519705.1 | d__Bacteria; p__Campylobacterota; c__Campylobacteria; o__Campylobacterales; f__Campylobacteraceae; g__Campylobacter_B; s__Campylobacter_B sp012519705 | 52.038500 | 8.495600 | anaerobic digestion of organic wastes under variable temperature conditions and feedstocks | Engineered;Bioreactor;Anaerobic;Digestate;Unclassified | 0.023% |
|  | GCA_012519705.1 | d__Bacteria; p__Campylobacterota; c__Campylobacteria; o__Campylobacterales; f__Campylobacteraceae; g__Campylobacter_B; s__Campylobacter_B sp012519705 | 52.038500 | 8.495600 | anaerobic digestion of organic wastes under variable temperature conditions and feedstocks | Engineered;Bioreactor;Anaerobic;Digestate;Unclassified | 0.021% |
| 11 | GCA_012521935.1 | d__Bacteria; p__Campylobacterota; c__Campylobacteria; o__Campylobacterales; f__UBA1877; g__JAAYLJ01; s__JAAYLJ01 sp012521935 | 55.788290 | 12.526820 | anaerobic digestion of organic wastes under variable temperature conditions and feedstocks | Engineered;Bioreactor;Anaerobic;Digestate;Unclassified | 0.131% |
|  | GCA_012521935.1 | d__Bacteria; p__Campylobacterota; c__Campylobacteria; o__Campylobacterales; f__UBA1877; g__JAAYLJ01; s__JAAYLJ01 sp012521935 | 55.788290 | 12.526820 | anaerobic digestion of organic wastes under variable temperature conditions and feedstocks | Engineered;Bioreactor;Anaerobic;Digestate;Unclassified | 0.129% |
|  | GCA_012521935.1 | d__Bacteria; p__Campylobacterota; c__Campylobacteria; o__Campylobacterales; f__UBA1877; g__JAAYLJ01; s__JAAYLJ01 sp012521935 | 55.788290 | 12.526820 | anaerobic digestion of organic wastes under variable temperature conditions and feedstocks | Engineered;Bioreactor;Anaerobic;Digestate;Unclassified | 0.160% |
|  | GCA_012521935.1 | d__Bacteria; p__Campylobacterota; c__Campylobacteria; o__Campylobacterales; f__UBA1877; g__JAAYLJ01; s__JAAYLJ01 sp012521935 | 55.788290 | 12.526820 | anaerobic digestion of organic wastes under variable temperature conditions and feedstocks | Engineered;Bioreactor;Anaerobic;Digestate;Unclassified | 0.234% |
| 12 | GCA_012728225.1 | d__Bacteria; p__Campylobacterota; c__Campylobacteria; o__Campylobacterales; f__Campylobacteraceae; g__Campylobacter_B; s__Campylobacter_B sp012728225 | 55.788335 | 12.526673 | anaerobic digestion of organic wastes under variable temperature conditions and feedstocks | Engineered;Bioreactor;Anaerobic;Digestate;Unclassified | 0.153% |
|  | GCA_012728225.1 | d__Bacteria; p__Campylobacterota; c__Campylobacteria; o__Campylobacterales; f__Campylobacteraceae; g__Campylobacter_B; s__Campylobacter_B sp012728225 | 55.788335 | 12.526673 | anaerobic digestion of organic wastes under variable temperature conditions and feedstocks | Engineered;Bioreactor;Anaerobic;Digestate;Unclassified | 0.169% |
|  | GCA_012728225.1 | d__Bacteria; p__Campylobacterota; c__Campylobacteria; o__Campylobacterales; f__Campylobacteraceae; g__Campylobacter_B; s__Campylobacter_B sp012728225 | 55.788335 | 12.526673 | anaerobic digestion of organic wastes under variable temperature conditions and feedstocks | Engineered;Bioreactor;Anaerobic;Digestate;Unclassified | 0.230% |
|  | GCA_012728225.1 | d__Bacteria; p__Campylobacterota; c__Campylobacteria; o__Campylobacterales; f__Campylobacteraceae; g__Campylobacter_B; s__Campylobacter_B sp012728225 | 55.788335 | 12.526673 | anaerobic digestion of organic wastes under variable temperature conditions and feedstocks | Engineered;Bioreactor;Anaerobic;Digestate;Unclassified | 0.195% |
|  | GCA_012728225.1 | d__Bacteria; p__Campylobacterota; c__Campylobacteria; o__Campylobacterales; f__Campylobacteraceae; g__Campylobacter_B; s__Campylobacter_B sp012728225 | 55.788335 | 12.526673 | anaerobic digestion of organic wastes under variable temperature conditions and feedstocks | Engineered;Bioreactor;Anaerobic;Digestate;Unclassified | 0.212% |
|  | GCA_012728225.1 | d__Bacteria; p__Campylobacterota; c__Campylobacteria; o__Campylobacterales; f__Campylobacteraceae; g__Campylobacter_B; s__Campylobacter_B sp012728225 | 55.788335 | 12.526673 | anaerobic digestion of organic wastes under variable temperature conditions and feedstocks | Engineered;Bioreactor;Anaerobic;Digestate;Unclassified | 0.268% |
|  | GCA_012728225.1 | d__Bacteria; p__Campylobacterota; c__Campylobacteria; o__Campylobacterales; f__Campylobacteraceae; g__Campylobacter_B; s__Campylobacter_B sp012728225 | 55.788335 | 12.526673 | anaerobic digestion of organic wastes under variable temperature conditions and feedstocks | Engineered;Bioreactor;Anaerobic;Digestate;Unclassified | 0.186% |
|  | GCA_012728225.1 | d__Bacteria; p__Campylobacterota; c__Campylobacteria; o__Campylobacterales; f__Campylobacteraceae; g__Campylobacter_B; s__Campylobacter_B sp012728225 | 55.788335 | 12.526673 | anaerobic digestion of organic wastes under variable temperature conditions and feedstocks | Engineered;Bioreactor;Anaerobic;Digestate;Unclassified | 0.157% |
|  | GCA_012728225.1 | d__Bacteria; p__Campylobacterota; c__Campylobacteria; o__Campylobacterales; f__Campylobacteraceae; g__Campylobacter_B; s__Campylobacter_B sp012728225 | 55.788335 | 12.526673 | anaerobic digestion of organic wastes under variable temperature conditions and feedstocks | Engineered;Bioreactor;Anaerobic;Digestate;Unclassified | 0.187% |
| 13 | GCA_013138865.1 | d__Bacteria; p__Campylobacterota; c__Campylobacteria; o__Campylobacterales; f__Sulfurimonadaceae; g__GLR69; s__GLR69 sp013138865 | -41.688783 | 175.681183 | Glendhu Ridge methane seep | Environmental;Aquatic;Marine;Oceanic;Sediment | 0.005% |
|  | GCA_013138865.1 | d__Bacteria; p__Campylobacterota; c__Campylobacteria; o__Campylobacterales; f__Sulfurimonadaceae; g__GLR69; s__GLR69 sp013138865 | -41.770333 | 176.083500 | Glendhu Ridge methane seep | Environmental;Aquatic;Marine;Cold seeps;Sediment | 0.210% |
|  | GCA_013138865.1 | d__Bacteria; p__Campylobacterota; c__Campylobacteria; o__Campylobacterales; f__Sulfurimonadaceae; g__GLR69; s__GLR69 sp013138865 | -41.770333 | 176.083500 | Glendhu Ridge methane seep | Environmental;Aquatic;Marine;Cold seeps;Sediment | 0.026% |
|  | GCA_013138865.1 | d__Bacteria; p__Campylobacterota; c__Campylobacteria; o__Campylobacterales; f__Sulfurimonadaceae; g__GLR69; s__GLR69 sp013138865 | -41.688783 | 175.681183 | Glendhu Ridge methane seep | Engineered;Lab enrichment;Undefined media;Unclassified;Unclassified | 0.180% |
|  | GCA_013138865.1 | d__Bacteria; p__Campylobacterota; c__Campylobacteria; o__Campylobacterales; f__Sulfurimonadaceae; g__GLR69; s__GLR69 sp013138865 | -41.770333 | 176.083500 | Glendhu Ridge methane seep | Engineered;Lab enrichment;Undefined media;Unclassified;Unclassified | 0.036% |
|  | GCA_013138865.1 | d__Bacteria; p__Campylobacterota; c__Campylobacteria; o__Campylobacterales; f__Sulfurimonadaceae; g__GLR69; s__GLR69 sp013138865 | -41.770333 | 176.083500 | Glendhu Ridge methane seep | Engineered;Lab enrichment;Undefined media;Unclassified;Unclassified | 1.239% |
| 14 | GCA_013373175.1 | d__Bacteria; p__Campylobacterota; c__Campylobacteria; o__Campylobacterales; f__Arcobacteraceae; g__Halarcobacter; s__Halarcobacter sp013373175 | 31.417780 | -81.296041 | surface seawater inoculated into a synthetic phycosphere | Host-associated;Algae;Diatoms;Phycosphere;Unclassified | - |
| 15 | GCA_014381585.1 | d__Bacteria; p__Campylobacterota; c__Campylobacteria; o__Campylobacterales; f__Sulfurimonadaceae; g__Sulfurimonas; s__Sulfurimonas ponti | 42.896667 | 30.678333 | Suspended Particulate Matter (SPM) from the Black Sea western gyre | Environmental;Aquatic;Marine;Oceanic;Unclassified | 0.481% |
|  | GCA_014381585.1 | d__Bacteria; p__Campylobacterota; c__Campylobacteria; o__Campylobacterales; f__Sulfurimonadaceae; g__Sulfurimonas; s__Sulfurimonas ponti | 42.896667 | 30.678333 | Suspended Particulate Matter (SPM) from the Black Sea western gyre | Environmental;Aquatic;Marine;Oceanic;Unclassified | 0.102% |
|  | GCA_014381585.1 | d__Bacteria; p__Campylobacterota; c__Campylobacteria; o__Campylobacterales; f__Sulfurimonadaceae; g__Sulfurimonas; s__Sulfurimonas ponti | 42.896667 | 30.678333 | Suspended Particulate Matter (SPM) from the Black Sea western gyre | Environmental;Aquatic;Marine;Oceanic;Unclassified | 0.482% |
|  | GCA_014381585.1 | d__Bacteria; p__Campylobacterota; c__Campylobacteria; o__Campylobacterales; f__Sulfurimonadaceae; g__Sulfurimonas; s__Sulfurimonas ponti | 42.896667 | 30.678333 | Suspended Particulate Matter (SPM) from the Black Sea western gyre | Environmental;Aquatic;Marine;Oceanic;Unclassified | 0.186% |
|  | GCA_014381585.1 | d__Bacteria; p__Campylobacterota; c__Campylobacteria; o__Campylobacterales; f__Sulfurimonadaceae; g__Sulfurimonas; s__Sulfurimonas ponti | 42.896667 | 30.678333 | Suspended Particulate Matter (SPM) from the Black Sea western gyre | Environmental;Aquatic;Marine;Oceanic;Unclassified | 1.022% |
|  | GCA_014381585.1 | d__Bacteria; p__Campylobacterota; c__Campylobacteria; o__Campylobacterales; f__Sulfurimonadaceae; g__Sulfurimonas; s__Sulfurimonas ponti | 42.896667 | 30.678333 | Suspended Particulate Matter (SPM) from the Black Sea western gyre | Environmental;Aquatic;Marine;Oceanic;Unclassified | 1.017% |
|  | GCA_014381585.1 | d__Bacteria; p__Campylobacterota; c__Campylobacteria; o__Campylobacterales; f__Sulfurimonadaceae; g__Sulfurimonas; s__Sulfurimonas ponti | 42.896667 | 30.678333 | Suspended Particulate Matter (SPM) from the Black Sea western gyre | Environmental;Aquatic;Marine;Oceanic;Unclassified | 1.150% |
|  | GCA_014381585.1 | d__Bacteria; p__Campylobacterota; c__Campylobacteria; o__Campylobacterales; f__Sulfurimonadaceae; g__Sulfurimonas; s__Sulfurimonas ponti | 42.896667 | 30.678333 | Suspended Particulate Matter (SPM) from the Black Sea western gyre | Environmental;Aquatic;Marine;Oceanic;Unclassified | 1.725% |
|  | GCA_014381585.1 | d__Bacteria; p__Campylobacterota; c__Campylobacteria; o__Campylobacterales; f__Sulfurimonadaceae; g__Sulfurimonas; s__Sulfurimonas ponti | 42.896667 | 30.678333 | Suspended Particulate Matter (SPM) from the Black Sea western gyre | Environmental;Aquatic;Marine;Oceanic;Unclassified | 1.773% |
|  | GCA_014381585.1 | d__Bacteria; p__Campylobacterota; c__Campylobacteria; o__Campylobacterales; f__Sulfurimonadaceae; g__Sulfurimonas; s__Sulfurimonas ponti | 42.896667 | 30.678333 | Suspended Particulate Matter (SPM) from the Black Sea western gyre | Environmental;Aquatic;Marine;Oceanic;Unclassified | 2.240% |
|  | GCA_014381585.1 | d__Bacteria; p__Campylobacterota; c__Campylobacteria; o__Campylobacterales; f__Sulfurimonadaceae; g__Sulfurimonas; s__Sulfurimonas ponti | 42.896667 | 30.678333 | Suspended Particulate Matter (SPM) from the Black Sea western gyre | Environmental;Aquatic;Marine;Oceanic;Unclassified | 2.620% |
|  | GCA_014381585.1 | d__Bacteria; p__Campylobacterota; c__Campylobacteria; o__Campylobacterales; f__Sulfurimonadaceae; g__Sulfurimonas; s__Sulfurimonas ponti | 42.896667 | 30.678333 | Suspended Particulate Matter (SPM) from the Black Sea western gyre | Environmental;Aquatic;Marine;Oceanic;Unclassified | 1.378% |
|  | GCA_014381585.1 | d__Bacteria; p__Campylobacterota; c__Campylobacteria; o__Campylobacterales; f__Sulfurimonadaceae; g__Sulfurimonas; s__Sulfurimonas ponti | 42.896667 | 30.678333 | Suspended Particulate Matter (SPM) from the Black Sea western gyre | Environmental;Aquatic;Marine;Oceanic;Unclassified | 0.895% |
|  | GCA_014381585.1 | d__Bacteria; p__Campylobacterota; c__Campylobacteria; o__Campylobacterales; f__Sulfurimonadaceae; g__Sulfurimonas; s__Sulfurimonas ponti | 42.896667 | 30.678333 | Suspended Particulate Matter (SPM) from the Black Sea western gyre | Environmental;Aquatic;Marine;Oceanic;Unclassified | 0.028% |
|  | GCA_014381585.1 | d__Bacteria; p__Campylobacterota; c__Campylobacteria; o__Campylobacterales; f__Sulfurimonadaceae; g__Sulfurimonas; s__Sulfurimonas ponti | 42.896667 | 30.678333 | Suspended Particulate Matter (SPM) from the Black Sea western gyre | Environmental;Aquatic;Marine;Oceanic;Unclassified | 0.010% |
| 16 | GCA_014859645.1 | d__Bacteria; p__Campylobacterota; c__Campylobacteria; o__Campylobacterales; f__UBA1877; g__Sulfurospirillum_B; s__Sulfurospirillum_B sp014859645 | 45.426100 | 36.478000 | bubbling pool in the central crater-like structure of an active mud volcano | Environmental;Aquatic;Marine;Mud volcano;Unclassified | 0.973% |
| 17 | GCA_014859685.1 | d__Bacteria; p__Campylobacterota; c__Campylobacteria; o__Campylobacterales; f__Sulfurospirillaceae; g__UBA6810; s__UBA6810 sp014859685 | 45.426100 | 36.478000 | bubbling pool in the central crater-like structure of an active mud volcano | Environmental;Aquatic;Marine;Mud volcano;Unclassified | 7.379% |
| 18 | GCA_015487435.1 | d__Bacteria; p__Campylobacterota; c__Campylobacteria; o__Campylobacterales; f__Sulfurimonadaceae; g__Sulfurimonas; s__Sulfurimonas sp015487435 | -34.857920 | 179.051933 | deep-sea hydrothermal deposit | Environmental;Aquatic;Marine;Hydrothermal vents;Unclassified | 1.253% |
| 19 | GCA_016744795.1 | d__Bacteria; p__Campylobacterota; c__Campylobacteria; o__Campylobacterales; f__Sulfurospirillaceae; g__Sulfurospirillum_A; s__Sulfurospirillum_A sp016744795 | 60.238185 | 5.181210 | biofilm scrapped from cow tibia surface after nine month of incubation underwater in a fjord | Host-associated;Mammals;Skeletal system;Bone;Unclassified | 6.271% |
|  | GCA_016744795.1 | d__Bacteria; p__Campylobacterota; c__Campylobacteria; o__Campylobacterales; f__Sulfurospirillaceae; g__Sulfurospirillum_A; s__Sulfurospirillum_A sp016744795 | 60.238185 | 5.181210 | biofilm scrapped from cow tibia surface after nine month of incubation underwater in a fjord | Host-associated;Mammals;Skeletal system;Bone;Unclassified | 0.599% |
|  | GCA_016744795.1 | d__Bacteria; p__Campylobacterota; c__Campylobacteria; o__Campylobacterales; f__Sulfurospirillaceae; g__Sulfurospirillum_A; s__Sulfurospirillum_A sp016744795 | 60.238185 | 5.181210 | biofilm scrapped from cow tibia surface after nine month of incubation underwater in a fjord | Host-associated;Mammals;Skeletal system;Bone;Unclassified | 1.088% |
|  | GCA_016744795.1 | d__Bacteria; p__Campylobacterota; c__Campylobacteria; o__Campylobacterales; f__Sulfurospirillaceae; g__Sulfurospirillum_A; s__Sulfurospirillum_A sp016744795 | 60.238185 | 5.181210 | biofilm scrapped from cow tibia surface after nine month of incubation underwater in a fjord | Host-associated;Mammals;Skeletal system;Bone;Unclassified | 0.521% |
| 20 | GCA_016744875.1 | d__Bacteria; p__Campylobacterota; c__Campylobacteria; o__Campylobacterales; f__Sulfurovaceae; g__Sulfurovum; s__Sulfurovum sp016744875 | 60.238185 | 5.181210 | biofilm scrapped from cow tibia surface after nine month of incubation underwater in a fjord | Host-associated;Mammals;Skeletal system;Bone;Unclassified | 0.328% |
|  | GCA_016744875.1 | d__Bacteria; p__Campylobacterota; c__Campylobacteria; o__Campylobacterales; f__Sulfurovaceae; g__Sulfurovum; s__Sulfurovum sp016744875 | 60.238185 | 5.181210 | biofilm scrapped from cow tibia surface after nine month of incubation underwater in a fjord | Host-associated;Mammals;Skeletal system;Bone;Unclassified | 0.315% |
|  | GCA_016744875.1 | d__Bacteria; p__Campylobacterota; c__Campylobacteria; o__Campylobacterales; f__Sulfurovaceae; g__Sulfurovum; s__Sulfurovum sp016744875 | 60.238185 | 5.181210 | biofilm scrapped from cow tibia surface after nine month of incubation underwater in a fjord | Host-associated;Mammals;Skeletal system;Bone;Unclassified | 1.295% |
|  | GCA_016744875.1 | d__Bacteria; p__Campylobacterota; c__Campylobacteria; o__Campylobacterales; f__Sulfurovaceae; g__Sulfurovum; s__Sulfurovum sp016744875 | 60.238185 | 5.181210 | biofilm scrapped from cow tibia surface after nine month of incubation underwater in a fjord | Host-associated;Mammals;Skeletal system;Bone;Unclassified | 0.199% |
| 21 | GCA_016930915.1 | d__Bacteria; p__Campylobacterota; c__Campylobacteria; o__Campylobacterales; f__Sulfurospirillaceae; g__Sulfurospirillum; s__Sulfurospirillum sp016930915 | 34.995620 | -98.688950 | freshwater sediment | Environmental;Aquatic;Freshwater;Lake;Sediment | 0.028% |
| 22 | GCA_017860615.1 | d__Bacteria; p__Campylobacterota; c__Campylobacteria; o__Campylobacterales; f__Sulfurospirillaceae; g__Sulfurospirillum; s__Sulfurospirillum sp017860615 | 41.377700 | -82.511700 | wetland soil | Environmental;Aquatic;Freshwater;Wetlands;Soil | 0.777% |
| 23 | GCA_019115525.1 | d__Bacteria; p__Campylobacterota; c__Campylobacteria; o__Campylobacterales; f__Helicobacteraceae; g__Helicobacter_G; s__Helicobacter_G avistercoris | 42.733900 | 25.485800 | gut metagenome (*Gallus gallus*) | Host-associated;Birds;Digestive system;Unclassified;Unclassified | 0.446% |
| 24 | GCA_021206415.1 | d__Bacteria; p__Campylobacterota; c__Campylobacteria; o__Campylobacterales; f__Sulfurospirillaceae; g__Sulfurospirillum; s__Sulfurospirillum sp021206415 | 21.761524 | 70.627625 | Textile industry effluent | Engineered;Wastewater;Industrial wastewater;Unclassified;Unclassified | - |
| 25 | GCA_021647715.1 | d__Bacteria; p__Campylobacterota; c__Campylobacteria; o__Campylobacterales; f__Sulfurovaceae; g__Sulfurovum; s__Sulfurovum sp021647715 | -37.782760 | 49.649360 | microbes were collected at JL-Dive100-S03 | Environmental;Aquatic;Marine;Sediment;Unclassified | - |
| 26 | GCA_021648465.1 | d__Bacteria; p__Campylobacterota; c__Campylobacteria; o__Campylobacterales; f__Sulfurimonadaceae; g__Sulfurimonas; s__Sulfurimonas sp021648465 | -37.783294 | 49.649117 | microbes were collected at JL-Dive96-S01 | Environmental;Aquatic;Marine;Sediment;Unclassified | - |
| 27 | GCA_022511145.1 | d__Bacteria; p__Campylobacterota; c__Campylobacteria; o__Campylobacterales; f__Helicobacteraceae; g__Helicobacter_D; s__Helicobacter_D sp022511145 | 33.550000 | 97.600000 | gut metagenome (*Plateau pika*) | Host-associated;Mammals;Digestive system;Unclassified;Unclassified | 0.418% |
| 28 | GCA_022511325.1 | d__Bacteria; p__Campylobacterota; c__Campylobacteria; o__Campylobacterales; f__Campylobacteraceae; g__Campylobacter_D; s__Campylobacter_D sp022511325 | 33.550000 | 97.600000 | gut metagenome (*Plateau pika*) | Host-associated;Mammals;Digestive system;Unclassified;Unclassified | 0.581% |
| 29 | GCA_022775765.1 | d__Bacteria; p__Campylobacterota; c__Campylobacteria; o__Campylobacterales; f__Campylobacteraceae; g__Campylobacter_B; s__Campylobacter_B sp022775765 | 52.468500 | -113.730700 | pig gut metagenome (*Sus scrofa domesticus*) | Host-associated;Mammals;Digestive system;Unclassified;Unclassified | 1.057% |
| 30 | GCA_024680445.1 | d__Bacteria; p__Campylobacterota; c__Campylobacteria; o__Campylobacterales; f__Campylobacteraceae; g__Campylobacter_A; s__Campylobacter_A sp024680445 | 35.840000 | 50.939100 | camel gut metagenome | Host-associated;Mammals;Digestive system;Unclassified;Unclassified | 0.268% |
|  | GCA_024680445.1 | d__Bacteria; p__Campylobacterota; c__Campylobacteria; o__Campylobacterales; f__Campylobacteraceae; g__Campylobacter_A; s__Campylobacter_A sp024680445 | 35.840000 | 50.939100 | camel gut metagenome | Host-associated;Mammals;Digestive system;Unclassified;Unclassified | 0.136% |
|  | GCA_024680445.1 | d__Bacteria; p__Campylobacterota; c__Campylobacteria; o__Campylobacterales; f__Campylobacteraceae; g__Campylobacter_A; s__Campylobacter_A sp024680445 | 35.840000 | 50.939100 | camel gut metagenome | Host-associated;Mammals;Digestive system;Unclassified;Unclassified | 0.174% |
|  | GCA_024680445.1 | d__Bacteria; p__Campylobacterota; c__Campylobacteria; o__Campylobacterales; f__Campylobacteraceae; g__Campylobacter_A; s__Campylobacter_A sp024680445 | 35.840000 | 50.939100 | camel gut metagenome | Host-associated;Mammals;Digestive system;Unclassified;Unclassified | 0.180% |
|  | GCA_024680445.1 | d__Bacteria; p__Campylobacterota; c__Campylobacteria; o__Campylobacterales; f__Campylobacteraceae; g__Campylobacter_A; s__Campylobacter_A sp024680445 | 35.840000 | 50.939100 | camel gut metagenome | Host-associated;Mammals;Digestive system;Unclassified;Unclassified | 0.085% |
|  | GCA_024680445.1 | d__Bacteria; p__Campylobacterota; c__Campylobacteria; o__Campylobacterales; f__Campylobacteraceae; g__Campylobacter_A; s__Campylobacter_A sp024680445 | 35.840000 | 50.939100 | camel gut metagenome | Host-associated;Mammals;Digestive system;Unclassified;Unclassified | 0.130% |
|  | GCA_024680445.1 | d__Bacteria; p__Campylobacterota; c__Campylobacteria; o__Campylobacterales; f__Campylobacteraceae; g__Campylobacter_A; s__Campylobacter_A sp024680445 | 35.840000 | 50.939100 | camel gut metagenome | Host-associated;Mammals;Digestive system;Unclassified;Unclassified | 0.094% |
|  | GCA_024680445.1 | d__Bacteria; p__Campylobacterota; c__Campylobacteria; o__Campylobacterales; f__Campylobacteraceae; g__Campylobacter_A; s__Campylobacter_A sp024680445 | 35.840000 | 50.939100 | camel gut metagenome | Host-associated;Mammals;Digestive system;Unclassified;Unclassified | 0.107% |
|  | GCA_024680445.1 | d__Bacteria; p__Campylobacterota; c__Campylobacteria; o__Campylobacterales; f__Campylobacteraceae; g__Campylobacter_A; s__Campylobacter_A sp024680445 | 35.840000 | 50.939100 | camel gut metagenome | Host-associated;Mammals;Digestive system;Unclassified;Unclassified | 0.184% |
|  | GCA_024680445.1 | d__Bacteria; p__Campylobacterota; c__Campylobacteria; o__Campylobacterales; f__Campylobacteraceae; g__Campylobacter_A; s__Campylobacter_A sp024680445 | 35.840000 | 50.939100 | camel gut metagenome | Host-associated;Mammals;Digestive system;Unclassified;Unclassified | 0.103% |
|  | GCA_024680445.1 | d__Bacteria; p__Campylobacterota; c__Campylobacteria; o__Campylobacterales; f__Campylobacteraceae; g__Campylobacter_A; s__Campylobacter_A sp024680445 | 35.840000 | 50.939100 | camel gut metagenome | Host-associated;Mammals;Digestive system;Unclassified;Unclassified | 0.133% |
|  | GCA_024680445.1 | d__Bacteria; p__Campylobacterota; c__Campylobacteria; o__Campylobacterales; f__Campylobacteraceae; g__Campylobacter_A; s__Campylobacter_A sp024680445 | 35.840000 | 50.939100 | camel gut metagenome | Host-associated;Mammals;Digestive system;Unclassified;Unclassified | 0.124% |
|  | GCA_024680445.1 | d__Bacteria; p__Campylobacterota; c__Campylobacteria; o__Campylobacterales; f__Campylobacteraceae; g__Campylobacter_A; s__Campylobacter_A sp024680445 | 35.840000 | 50.939100 | camel gut metagenome | Host-associated;Mammals;Digestive system;Unclassified;Unclassified | 0.157% |
|  | GCA_024680445.1 | d__Bacteria; p__Campylobacterota; c__Campylobacteria; o__Campylobacterales; f__Campylobacteraceae; g__Campylobacter_A; s__Campylobacter_A sp024680445 | 35.840000 | 50.939100 | camel gut metagenome | Host-associated;Mammals;Digestive system;Unclassified;Unclassified | 0.199% |
|  | GCA_024680445.1 | d__Bacteria; p__Campylobacterota; c__Campylobacteria; o__Campylobacterales; f__Campylobacteraceae; g__Campylobacter_A; s__Campylobacter_A sp024680445 | 35.840000 | 50.939100 | camel gut metagenome | Host-associated;Mammals;Digestive system;Unclassified;Unclassified | 0.188% |
|  | GCA_024680445.1 | d__Bacteria; p__Campylobacterota; c__Campylobacteria; o__Campylobacterales; f__Campylobacteraceae; g__Campylobacter_A; s__Campylobacter_A sp024680445 | 35.840000 | 50.939100 | camel gut metagenome | Host-associated;Mammals;Digestive system;Unclassified;Unclassified | 0.185% |
|  | GCA_024680445.1 | d__Bacteria; p__Campylobacterota; c__Campylobacteria; o__Campylobacterales; f__Campylobacteraceae; g__Campylobacter_A; s__Campylobacter_A sp024680445 | 35.840000 | 50.939100 | camel gut metagenome | Host-associated;Mammals;Digestive system;Unclassified;Unclassified | 0.323% |
|  | GCA_024680445.1 | d__Bacteria; p__Campylobacterota; c__Campylobacteria; o__Campylobacterales; f__Campylobacteraceae; g__Campylobacter_A; s__Campylobacter_A sp024680445 | 35.840000 | 50.939100 | camel gut metagenome | Host-associated;Mammals;Digestive system;Unclassified;Unclassified | 0.151% |
|  | GCA_024680445.1 | d__Bacteria; p__Campylobacterota; c__Campylobacteria; o__Campylobacterales; f__Campylobacteraceae; g__Campylobacter_A; s__Campylobacter_A sp024680445 | 35.840000 | 50.939100 | camel gut metagenome | Host-associated;Mammals;Digestive system;Unclassified;Unclassified | 0.142% |
|  | GCA_024680445.1 | d__Bacteria; p__Campylobacterota; c__Campylobacteria; o__Campylobacterales; f__Campylobacteraceae; g__Campylobacter_A; s__Campylobacter_A sp024680445 | 35.840000 | 50.939100 | camel gut metagenome | Host-associated;Mammals;Digestive system;Unclassified;Unclassified | 0.165% |
|  | GCA_024680445.1 | d__Bacteria; p__Campylobacterota; c__Campylobacteria; o__Campylobacterales; f__Campylobacteraceae; g__Campylobacter_A; s__Campylobacter_A sp024680445 | 35.840000 | 50.939100 | camel gut metagenome | Host-associated;Mammals;Digestive system;Unclassified;Unclassified | 0.081% |
|  | GCA_024680445.1 | d__Bacteria; p__Campylobacterota; c__Campylobacteria; o__Campylobacterales; f__Campylobacteraceae; g__Campylobacter_A; s__Campylobacter_A sp024680445 | 35.840000 | 50.939100 | camel gut metagenome | Host-associated;Mammals;Digestive system;Unclassified;Unclassified | 0.081% |
|  | GCA_024680445.1 | d__Bacteria; p__Campylobacterota; c__Campylobacteria; o__Campylobacterales; f__Campylobacteraceae; g__Campylobacter_A; s__Campylobacter_A sp024680445 | 35.840000 | 50.939100 | camel gut metagenome | Host-associated;Mammals;Digestive system;Unclassified;Unclassified | 0.152% |
|  | GCA_024680445.1 | d__Bacteria; p__Campylobacterota; c__Campylobacteria; o__Campylobacterales; f__Campylobacteraceae; g__Campylobacter_A; s__Campylobacter_A sp024680445 | 35.840000 | 50.939100 | camel gut metagenome | Host-associated;Mammals;Digestive system;Unclassified;Unclassified | 0.155% |
|  | GCA_024680445.1 | d__Bacteria; p__Campylobacterota; c__Campylobacteria; o__Campylobacterales; f__Campylobacteraceae; g__Campylobacter_A; s__Campylobacter_A sp024680445 | 35.840000 | 50.939100 | camel gut metagenome | Host-associated;Mammals;Digestive system;Unclassified;Unclassified | 0.120% |
|  | GCA_024680445.1 | d__Bacteria; p__Campylobacterota; c__Campylobacteria; o__Campylobacterales; f__Campylobacteraceae; g__Campylobacter_A; s__Campylobacter_A sp024680445 | 35.840000 | 50.939100 | camel gut metagenome | Host-associated;Mammals;Digestive system;Unclassified;Unclassified | 0.078% |
|  | GCA_024680445.1 | d__Bacteria; p__Campylobacterota; c__Campylobacteria; o__Campylobacterales; f__Campylobacteraceae; g__Campylobacter_A; s__Campylobacter_A sp024680445 | 35.840000 | 50.939100 | camel gut metagenome | Host-associated;Mammals;Digestive system;Unclassified;Unclassified | 0.098% |
|  | GCA_024680445.1 | d__Bacteria; p__Campylobacterota; c__Campylobacteria; o__Campylobacterales; f__Campylobacteraceae; g__Campylobacter_A; s__Campylobacter_A sp024680445 | 35.840000 | 50.939100 | camel gut metagenome | Host-associated;Mammals;Digestive system;Unclassified;Unclassified | 0.154% |
|  | GCA_024680445.1 | d__Bacteria; p__Campylobacterota; c__Campylobacteria; o__Campylobacterales; f__Campylobacteraceae; g__Campylobacter_A; s__Campylobacter_A sp024680445 | 35.840000 | 50.939100 | camel gut metagenome | Host-associated;Mammals;Digestive system;Unclassified;Unclassified | 0.164% |
|  | GCA_024680445.1 | d__Bacteria; p__Campylobacterota; c__Campylobacteria; o__Campylobacterales; f__Campylobacteraceae; g__Campylobacter_A; s__Campylobacter_A sp024680445 | 35.840000 | 50.939100 | camel gut metagenome | Host-associated;Mammals;Digestive system;Unclassified;Unclassified | 0.154% |
|  | GCA_024680445.1 | d__Bacteria; p__Campylobacterota; c__Campylobacteria; o__Campylobacterales; f__Campylobacteraceae; g__Campylobacter_A; s__Campylobacter_A sp024680445 | 35.840000 | 50.939100 | camel gut metagenome | Host-associated;Mammals;Digestive system;Unclassified;Unclassified | 0.099% |
|  | GCA_024680445.1 | d__Bacteria; p__Campylobacterota; c__Campylobacteria; o__Campylobacterales; f__Campylobacteraceae; g__Campylobacter_A; s__Campylobacter_A sp024680445 | 35.840000 | 50.939100 | camel gut metagenome | Host-associated;Mammals;Digestive system;Unclassified;Unclassified | 0.160% |
|  | GCA_024680445.1 | d__Bacteria; p__Campylobacterota; c__Campylobacteria; o__Campylobacterales; f__Campylobacteraceae; g__Campylobacter_A; s__Campylobacter_A sp024680445 | 35.840000 | 50.939100 | camel gut metagenome | Host-associated;Mammals;Digestive system;Unclassified;Unclassified | 0.098% |
|  | GCA_024680445.1 | d__Bacteria; p__Campylobacterota; c__Campylobacteria; o__Campylobacterales; f__Campylobacteraceae; g__Campylobacter_A; s__Campylobacter_A sp024680445 | 35.840000 | 50.939100 | camel gut metagenome | Host-associated;Mammals;Digestive system;Unclassified;Unclassified | 0.092% |
|  | GCA_024680445.1 | d__Bacteria; p__Campylobacterota; c__Campylobacteria; o__Campylobacterales; f__Campylobacteraceae; g__Campylobacter_A; s__Campylobacter_A sp024680445 | 35.840000 | 50.939100 | camel gut metagenome | Host-associated;Mammals;Digestive system;Unclassified;Unclassified | 0.183% |
|  | GCA_024680445.1 | d__Bacteria; p__Campylobacterota; c__Campylobacteria; o__Campylobacterales; f__Campylobacteraceae; g__Campylobacter_A; s__Campylobacter_A sp024680445 | 35.840000 | 50.939100 | camel gut metagenome | Host-associated;Mammals;Digestive system;Unclassified;Unclassified | 0.171% |
|  | GCA_024680445.1 | d__Bacteria; p__Campylobacterota; c__Campylobacteria; o__Campylobacterales; f__Campylobacteraceae; g__Campylobacter_A; s__Campylobacter_A sp024680445 | 35.840000 | 50.939100 | camel gut metagenome | Host-associated;Mammals;Digestive system;Unclassified;Unclassified | 0.264% |
|  | GCA_024680445.1 | d__Bacteria; p__Campylobacterota; c__Campylobacteria; o__Campylobacterales; f__Campylobacteraceae; g__Campylobacter_A; s__Campylobacter_A sp024680445 | 35.840000 | 50.939100 | camel gut metagenome | Host-associated;Mammals;Digestive system;Unclassified;Unclassified | 0.254% |
|  | GCA_024680445.1 | d__Bacteria; p__Campylobacterota; c__Campylobacteria; o__Campylobacterales; f__Campylobacteraceae; g__Campylobacter_A; s__Campylobacter_A sp024680445 | 35.840000 | 50.939100 | camel gut metagenome | Host-associated;Mammals;Digestive system;Unclassified;Unclassified | 0.281% |
|  | GCA_024680445.1 | d__Bacteria; p__Campylobacterota; c__Campylobacteria; o__Campylobacterales; f__Campylobacteraceae; g__Campylobacter_A; s__Campylobacter_A sp024680445 | 35.840000 | 50.939100 | camel gut metagenome | Host-associated;Mammals;Digestive system;Unclassified;Unclassified | 0.302% |
|  | GCA_024680445.1 | d__Bacteria; p__Campylobacterota; c__Campylobacteria; o__Campylobacterales; f__Campylobacteraceae; g__Campylobacter_A; s__Campylobacter_A sp024680445 | 35.840000 | 50.939100 | camel gut metagenome | Host-associated;Mammals;Digestive system;Unclassified;Unclassified | 0.233% |
|  | GCA_024680445.1 | d__Bacteria; p__Campylobacterota; c__Campylobacteria; o__Campylobacterales; f__Campylobacteraceae; g__Campylobacter_A; s__Campylobacter_A sp024680445 | 35.840000 | 50.939100 | camel gut metagenome | Host-associated;Mammals;Digestive system;Unclassified;Unclassified | 0.105% |
|  | GCA_024680445.1 | d__Bacteria; p__Campylobacterota; c__Campylobacteria; o__Campylobacterales; f__Campylobacteraceae; g__Campylobacter_A; s__Campylobacter_A sp024680445 | 35.840000 | 50.939100 | camel gut metagenome | Host-associated;Mammals;Digestive system;Unclassified;Unclassified | 0.108% |
|  | GCA_024680445.1 | d__Bacteria; p__Campylobacterota; c__Campylobacteria; o__Campylobacterales; f__Campylobacteraceae; g__Campylobacter_A; s__Campylobacter_A sp024680445 | 35.840000 | 50.939100 | camel gut metagenome | Host-associated;Mammals;Digestive system;Unclassified;Unclassified | 0.121% |
| 31 | GCA_026982075.1 | d__Bacteria; p__Campylobacterota; c__Campylobacteria; o__Campylobacterales; f__Sulfurovaceae; g__Sulfurovum; s__Sulfurovum sp026982075 | 36.229700 | -33.901100 | marine hydrothermal vent | Environmental;Aquatic;Marine;Hydrothermal vents;Unclassified | 3.822% |
| 32 | GCA_026988125.1 | d__Bacteria; p__Campylobacterota; c__Campylobacteria; o__Campylobacterales; f__Sulfurospirillaceae; g__Sulfurospirillum_A; s__Sulfurospirillum_A sp026988125 | 36.229400 | -33.902800 | marine hydrothermal vent | Environmental;Aquatic;Marine;Hydrothermal vents;Unclassified | 2.021% |
| 33 | GCA_026988315.1 | d__Bacteria; p__Campylobacterota; c__Campylobacteria; o__Campylobacterales; f__Sulfurimonadaceae; g__Sulfurimonas; s__Sulfurimonas sp026988315 | 36.229400 | -33.902800 | marine hydrothermal vent | Environmental;Aquatic;Marine;Hydrothermal vents;Unclassified | 3.238% |
| 34 | GCA_027063305.1 | d__Bacteria; p__Campylobacterota; c__Campylobacteria; o__Campylobacterales; f__Sulfurovaceae; g__Sulfurovum; s__Sulfurovum sp027063305 | 27.007800 | -111.407100 | marine hydrothermal vent | Environmental;Aquatic;Marine;Hydrothermal vents;Unclassified | 1.331% |
| 35 | GCA_027064065.1 | d__Bacteria; p__Campylobacterota_A; c__Desulfurellia; o__Desulfurellales; f__Hippeaceae; g__S144-51; s__S144-51 sp027064065 | 27.006700 | -111.409400 | marine hydrothermal vent | Environmental;Aquatic;Marine;Hydrothermal vents;Unclassified | 1.434% |
| 36 | GCA_028684065.1 | d__Bacteria; p__Campylobacterota; c__Campylobacteria; o__Campylobacterales; f__Sulfurospirillaceae; g__Sulfurospirillum; s__Sulfurospirillum sp028684065 | 38.960000 | -75.670000 | municipal landfill leachate | Engineered;Wastewater;Industrial wastewater;Landfill leachate;Unclassified | 3.714% |
| 37 | GCA_028705785.1 | d__Bacteria; p__Campylobacterota; c__Campylobacteria; o__Campylobacterales; f__Sulfurospirillaceae; g__Sulfurospirillum; s__Sulfurospirillum sp028705785 | 38.960000 | -75.670000 | municipal landfill leachate | Engineered;Wastewater;Industrial wastewater;Landfill leachate;Unclassified | 0.258% |
| 38 | GCA_030145305.1 | d__Bacteria; p__Campylobacterota; c__Campylobacteria; o__Campylobacterales; f__Arcobacteraceae; g__Halarcobacter; s__Halarcobacter sp030145305 | 54.089720 | 7.967800 | marine sediment | Environmental;Aquatic;Marine;Coastal;Sediment | - |
| 39 | GCA_900539255.1 | d__Bacteria; p__Campylobacterota; c__Campylobacteria; o__Campylobacterales; f__Campylobacteraceae; g__Campylobacter_D; s__Campylobacter_D sp900539255 | -5.680000 | -73.330000 | human gut | Host-associated;Mammals: Human;Digestive system;Unclassified;Unclassified | 1.579% |
| 40 | GCA_900772495.1 | d__Bacteria; p__Campylobacterota; c__Campylobacteria; o__Campylobacterales; f__Campylobacteraceae; g__Campylobacter_D; s__Campylobacter_D sp900772495 | -5.680000 | -73.330000 | human gut | Host-associated;Mammals: Human;Digestive system;Unclassified;Unclassified | 0.637% |
| 41 | GCA_910578285.1 | d__Bacteria; p__Campylobacterota; c__Campylobacteria; o__Campylobacterales; f__Helicobacteraceae; g__NHYN01; s__NHYN01 sp910578285 | 39.888650 | 116.383510 | mouse gut | Host-associated;Mammals;Digestive system;Unclassified;Unclassified | 1.484% |
| 42 | GCA_934668765.1 | d__Bacteria; p__Campylobacterota; c__Campylobacteria; o__Campylobacterales; f__Helicobacteraceae; g__NHYM01; s__NHYM01 sp934668765 | -3.550000 | 34.580000 | human feces | Host-associated;Mammals: Human;Digestive system;Unclassified;Unclassified | 0.838% |
| 43 | GCA_934718785.1 | d__Bacteria; p__Campylobacterota; c__Campylobacteria; o__Campylobacterales; f__Campylobacteraceae; g__Campylobacter_D; s__Campylobacter_D sp934718785 | 28.170000 | 84.250000 | human feces | Host-associated;Mammals: Human;Digestive system;Unclassified;Unclassified | 0.138% |
| 44 | GCA_934728065.1 | d__Bacteria; p__Campylobacterota; c__Campylobacteria; o__Campylobacterales; f__Campylobacteraceae; g__Campylobacter_D; s__Campylobacter_D sp934728065 | -3.550000 | 34.580000 | human feces | Host-associated;Mammals: Human;Digestive system;Unclassified;Unclassified | 1.345% |
| 45 | GCA_937875715.1 | d__Bacteria; p__Campylobacterota; c__Campylobacteria; o__Campylobacterales; f__Campylobacteraceae; g__Campylobacter; s__Campylobacter sp937875715 | 42.250000 | 113.830000 | gut metagenome | Host-associated;Mammals;Digestive system;Unclassified;Unclassified | 0.297% |
| 46 | GCA_944323755.1 | d__Bacteria; p__Campylobacterota; c__Campylobacteria; o__Campylobacterales; f__Helicobacteraceae; g__NHYM01; s__NHYM01 sp944323755 | 48.858300 | 2.292300 | chicken caecal content | Host-associated;Birds;Digestive system;Unclassified;Unclassified | 0.515% |
| 47 | GCA_944323885.1 | d__Bacteria; p__Campylobacterota; c__Campylobacteria; o__Campylobacterales; f__Helicobacteraceae; g__NHYM01; s__NHYM01 sp944323885 | 48.858300 | 2.292300 | chicken caecal content | Host-associated;Birds;Digestive system;Unclassified;Unclassified | 0.307% |
| 48 | GCA_944325775.1 | d__Bacteria; p__Campylobacterota; c__Campylobacteria; o__Campylobacterales; f__Helicobacteraceae; g__Helicobacter_F; s__Helicobacter_F sp944325775 | 48.858300 | 2.292300 | chicken caecal content | Host-associated;Birds;Digestive system;Unclassified;Unclassified | 0.230% |
| 49 | GCA_944326445.1 | d__Bacteria; p__Campylobacterota; c__Campylobacteria; o__Campylobacterales; f__Helicobacteraceae; g__Helicobacter_F; s__Helicobacter_F sp944326445 | 48.858300 | 2.292300 | chicken caecal content | Host-associated;Birds;Digestive system;Unclassified;Unclassified | 0.241% |
| 50 | GCA_947176375.1 | d__Bacteria; p__Campylobacterota; c__Campylobacteria; o__Campylobacterales; f__Helicobacteraceae; g__NHYM01; s__NHYM01 sp947176375 | 56.180000 | -2.550000 | mouse gut metagenome | Host-associated;Mammals;Digestive system;Unclassified;Unclassified | 23.760% |
| 51 | GCA_947177485.1 | d__Bacteria; p__Campylobacterota; c__Campylobacteria; o__Campylobacterales; f__Helicobacteraceae; g__NHYN01; s__NHYN01 sp947177485 | 54.690000 | 25.230000 | mouse gut metagenome | Host-associated;Mammals;Digestive system;Unclassified;Unclassified | 1.770% |
| 52 | GCA_947494995.1 | d__Bacteria; p__Campylobacterota; c__Campylobacteria; o__Campylobacterales; f__Arcobacteraceae; g__Halarcobacter; s__Halarcobacter sp947494995 | 37.562000 | 122.121000 | marine macroalgae | Host-associated;Algae;Unclassified;Unclassified;Unclassified | - |
| 53 | GCA_947588965.1 | d__Bacteria; p__Campylobacterota; c__Campylobacteria; o__Campylobacterales; f__Helicobacteraceae; g__Helicobacter_D; s__Helicobacter_D sp947588965 | 43.527378 | -1.499673 | rabbit gut | Host-associated;Mammals;Digestive system;Unclassified;Unclassified | 0.220% |
|  | GCA_947588965.1 | d__Bacteria; p__Campylobacterota; c__Campylobacteria; o__Campylobacterales; f__Helicobacteraceae; g__Helicobacter_D; s__Helicobacter_D sp947588965 | 43.527378 | -1.499673 | rabbit gut | Host-associated;Mammals;Digestive system;Unclassified;Unclassified | 0.220% |
|  | GCA_947588965.1 | d__Bacteria; p__Campylobacterota; c__Campylobacteria; o__Campylobacterales; f__Helicobacteraceae; g__Helicobacter_D; s__Helicobacter_D sp947588965 | 43.527378 | -1.499673 | rabbit gut | Host-associated;Mammals;Digestive system;Unclassified;Unclassified | 0.220% |
|  | GCA_947588965.1 | d__Bacteria; p__Campylobacterota; c__Campylobacteria; o__Campylobacterales; f__Helicobacteraceae; g__Helicobacter_D; s__Helicobacter_D sp947588965 | 43.527378 | -1.499673 | rabbit gut | Host-associated;Mammals;Digestive system;Unclassified;Unclassified | 0.223% |
| 54 | GCA_949298825.1 | d__Bacteria; p__Campylobacterota; c__Campylobacteria; o__Campylobacterales; f__Helicobacteraceae; g__Helicobacter_H; s__Helicobacter_H sp949298825 | 11.160000 | 39.920000 | Caecal contents (*Gallus gallus*) | Host-associated;Birds;Digestive system;Unclassified;Unclassified | 0.309% |
| 55 | GCF_000743525.1 | d__Bacteria; p__Campylobacterota; c__Campylobacteria; o__Campylobacterales; f__Sulfurospirillaceae; g__Sulfurospirillum; s__Sulfurospirillum sp000743525 | 57.076388 | -111.638632 | mature fine tailings from oil sands tailings pond grown in methanogenic alkane degrading enrichment culture | Engineered;Built environment;Tailings pond;Wastewater;Unclassified | - |
| 56 | GCF_014799995.1 | d__Bacteria; p__Campylobacterota; c__Campylobacteria; o__Campylobacterales; f__Helicobacteraceae; g__Helicobacter_D; s__Helicobacter_D sp014799995 | 51.300000 | 30.070000 | gut metagenome (*Myodes glareolus*) | Host-associated;Mammals;Digestive system;Unclassified;Unclassified | 0.163% |
|  | GCF_014799995.1 | d__Bacteria; p__Campylobacterota; c__Campylobacteria; o__Campylobacterales; f__Helicobacteraceae; g__Helicobacter_D; s__Helicobacter_D sp014799995 | 51.300000 | 30.070000 | gut metagenome (*Myodes glareolus*) | Host-associated;Mammals;Digestive system;Unclassified;Unclassified | 0.148% |
|  | GCF_014799995.1 | d__Bacteria; p__Campylobacterota; c__Campylobacteria; o__Campylobacterales; f__Helicobacteraceae; g__Helicobacter_D; s__Helicobacter_D sp014799995 | 51.300000 | 30.070000 | gut metagenome (*Myodes glareolus*) | Host-associated;Mammals;Digestive system;Unclassified;Unclassified | 0.202% |
|  | GCF_014799995.1 | d__Bacteria; p__Campylobacterota; c__Campylobacteria; o__Campylobacterales; f__Helicobacteraceae; g__Helicobacter_D; s__Helicobacter_D sp014799995 | 51.300000 | 30.070000 | gut metagenome (*Myodes glareolus*) | Host-associated;Mammals;Digestive system;Unclassified;Unclassified | 0.148% |
|  | GCF_014799995.1 | d__Bacteria; p__Campylobacterota; c__Campylobacteria; o__Campylobacterales; f__Helicobacteraceae; g__Helicobacter_D; s__Helicobacter_D sp014799995 | 51.300000 | 30.070000 | gut metagenome (*Myodes glareolus*) | Host-associated;Mammals;Digestive system;Unclassified;Unclassified | 0.145% |
|  | GCF_014799995.1 | d__Bacteria; p__Campylobacterota; c__Campylobacteria; o__Campylobacterales; f__Helicobacteraceae; g__Helicobacter_D; s__Helicobacter_D sp014799995 | 51.300000 | 30.070000 | gut metagenome (*Myodes glareolus*) | Host-associated;Mammals;Digestive system;Unclassified;Unclassified | 0.104% |
|  | GCF_014799995.1 | d__Bacteria; p__Campylobacterota; c__Campylobacteria; o__Campylobacterales; f__Helicobacteraceae; g__Helicobacter_D; s__Helicobacter_D sp014799995 | 51.300000 | 30.070000 | gut metagenome (*Myodes glareolus*) | Host-associated;Mammals;Digestive system;Unclassified;Unclassified | 0.341% |
|  | GCF_014799995.1 | d__Bacteria; p__Campylobacterota; c__Campylobacteria; o__Campylobacterales; f__Helicobacteraceae; g__Helicobacter_D; s__Helicobacter_D sp014799995 | 51.300000 | 30.070000 | gut metagenome (*Myodes glareolus*) | Host-associated;Mammals;Digestive system;Unclassified;Unclassified | 0.153% |
|  | GCF_014799995.1 | d__Bacteria; p__Campylobacterota; c__Campylobacteria; o__Campylobacterales; f__Helicobacteraceae; g__Helicobacter_D; s__Helicobacter_D sp014799995 | 51.300000 | 30.070000 | gut metagenome (*Myodes glareolus*) | Host-associated;Mammals;Digestive system;Unclassified;Unclassified | 0.193% |
|  | GCF_014799995.1 | d__Bacteria; p__Campylobacterota; c__Campylobacteria; o__Campylobacterales; f__Helicobacteraceae; g__Helicobacter_D; s__Helicobacter_D sp014799995 | 51.300000 | 30.070000 | gut metagenome (*Myodes glareolus*) | Host-associated;Mammals;Digestive system;Unclassified;Unclassified | 0.153% |
|  | GCF_014799995.1 | d__Bacteria; p__Campylobacterota; c__Campylobacteria; o__Campylobacterales; f__Helicobacteraceae; g__Helicobacter_D; s__Helicobacter_D sp014799995 | 51.300000 | 30.070000 | gut metagenome (*Myodes glareolus*) | Host-associated;Mammals;Digestive system;Unclassified;Unclassified | 0.140% |
|  | GCF_014799995.1 | d__Bacteria; p__Campylobacterota; c__Campylobacteria; o__Campylobacterales; f__Helicobacteraceae; g__Helicobacter_D; s__Helicobacter_D sp014799995 | 51.300000 | 30.070000 | gut metagenome (*Myodes glareolus*) | Host-associated;Mammals;Digestive system;Unclassified;Unclassified | 0.146% |
|  | GCF_014799995.1 | d__Bacteria; p__Campylobacterota; c__Campylobacteria; o__Campylobacterales; f__Helicobacteraceae; g__Helicobacter_D; s__Helicobacter_D sp014799995 | 51.300000 | 30.070000 | gut metagenome (*Myodes glareolus*) | Host-associated;Mammals;Digestive system;Unclassified;Unclassified | 0.141% |
|  | GCF_014799995.1 | d__Bacteria; p__Campylobacterota; c__Campylobacteria; o__Campylobacterales; f__Helicobacteraceae; g__Helicobacter_D; s__Helicobacter_D sp014799995 | 51.300000 | 30.070000 | gut metagenome (*Myodes glareolus*) | Host-associated;Mammals;Digestive system;Unclassified;Unclassified | 0.155% |
|  | GCF_014799995.1 | d__Bacteria; p__Campylobacterota; c__Campylobacteria; o__Campylobacterales; f__Helicobacteraceae; g__Helicobacter_D; s__Helicobacter_D sp014799995 | 51.300000 | 30.070000 | gut metagenome (*Myodes glareolus*) | Host-associated;Mammals;Digestive system;Unclassified;Unclassified | 0.177% |
|  | GCF_014799995.1 | d__Bacteria; p__Campylobacterota; c__Campylobacteria; o__Campylobacterales; f__Helicobacteraceae; g__Helicobacter_D; s__Helicobacter_D sp014799995 | 51.300000 | 30.070000 | gut metagenome (*Myodes glareolus*) | Host-associated;Mammals;Digestive system;Unclassified;Unclassified | 0.170% |
|  | GCF_014799995.1 | d__Bacteria; p__Campylobacterota; c__Campylobacteria; o__Campylobacterales; f__Helicobacteraceae; g__Helicobacter_D; s__Helicobacter_D sp014799995 | 51.300000 | 30.070000 | gut metagenome (*Myodes glareolus*) | Host-associated;Mammals;Digestive system;Unclassified;Unclassified | 0.172% |
|  | GCF_014799995.1 | d__Bacteria; p__Campylobacterota; c__Campylobacteria; o__Campylobacterales; f__Helicobacteraceae; g__Helicobacter_D; s__Helicobacter_D sp014799995 | 51.300000 | 30.070000 | gut metagenome (*Myodes glareolus*) | Host-associated;Mammals;Digestive system;Unclassified;Unclassified | 0.142% |
|  | GCF_014799995.1 | d__Bacteria; p__Campylobacterota; c__Campylobacteria; o__Campylobacterales; f__Helicobacteraceae; g__Helicobacter_D; s__Helicobacter_D sp014799995 | 51.300000 | 30.070000 | gut metagenome (*Myodes glareolus*) | Host-associated;Mammals;Digestive system;Unclassified;Unclassified | 0.164% |
|  | GCF_014799995.1 | d__Bacteria; p__Campylobacterota; c__Campylobacteria; o__Campylobacterales; f__Helicobacteraceae; g__Helicobacter_D; s__Helicobacter_D sp014799995 | 51.300000 | 30.070000 | gut metagenome (*Myodes glareolus*) | Host-associated;Mammals;Digestive system;Unclassified;Unclassified | 0.171% |
|  | GCF_014799995.1 | d__Bacteria; p__Campylobacterota; c__Campylobacteria; o__Campylobacterales; f__Helicobacteraceae; g__Helicobacter_D; s__Helicobacter_D sp014799995 | 51.300000 | 30.070000 | gut metagenome (*Myodes glareolus*) | Host-associated;Mammals;Digestive system;Unclassified;Unclassified | 0.149% |
|  | GCF_014799995.1 | d__Bacteria; p__Campylobacterota; c__Campylobacteria; o__Campylobacterales; f__Helicobacteraceae; g__Helicobacter_D; s__Helicobacter_D sp014799995 | 51.300000 | 30.070000 | gut metagenome (*Myodes glareolus*) | Host-associated;Mammals;Digestive system;Unclassified;Unclassified | 0.176% |
|  | GCF_014799995.1 | d__Bacteria; p__Campylobacterota; c__Campylobacteria; o__Campylobacterales; f__Helicobacteraceae; g__Helicobacter_D; s__Helicobacter_D sp014799995 | 51.300000 | 30.070000 | gut metagenome (*Myodes glareolus*) | Host-associated;Mammals;Digestive system;Unclassified;Unclassified | 0.179% |
|  | GCF_014799995.1 | d__Bacteria; p__Campylobacterota; c__Campylobacteria; o__Campylobacterales; f__Helicobacteraceae; g__Helicobacter_D; s__Helicobacter_D sp014799995 | 51.300000 | 30.070000 | gut metagenome (*Myodes glareolus*) | Host-associated;Mammals;Digestive system;Unclassified;Unclassified | 0.185% |
|  | GCF_014799995.1 | d__Bacteria; p__Campylobacterota; c__Campylobacteria; o__Campylobacterales; f__Helicobacteraceae; g__Helicobacter_D; s__Helicobacter_D sp014799995 | 51.300000 | 30.070000 | gut metagenome (*Myodes glareolus*) | Host-associated;Mammals;Digestive system;Unclassified;Unclassified | 0.209% |
|  | GCF_014799995.1 | d__Bacteria; p__Campylobacterota; c__Campylobacteria; o__Campylobacterales; f__Helicobacteraceae; g__Helicobacter_D; s__Helicobacter_D sp014799995 | 51.300000 | 30.070000 | gut metagenome (*Myodes glareolus*) | Host-associated;Mammals;Digestive system;Unclassified;Unclassified | 0.200% |
|  | GCF_014799995.1 | d__Bacteria; p__Campylobacterota; c__Campylobacteria; o__Campylobacterales; f__Helicobacteraceae; g__Helicobacter_D; s__Helicobacter_D sp014799995 | 51.300000 | 30.070000 | gut metagenome (*Myodes glareolus*) | Host-associated;Mammals;Digestive system;Unclassified;Unclassified | 1.282% |
|  | GCF_014799995.1 | d__Bacteria; p__Campylobacterota; c__Campylobacteria; o__Campylobacterales; f__Helicobacteraceae; g__Helicobacter_D; s__Helicobacter_D sp014799995 | 51.300000 | 30.070000 | gut metagenome (*Myodes glareolus*) | Host-associated;Mammals;Digestive system;Unclassified;Unclassified | 0.147% |
|  | GCF_014799995.1 | d__Bacteria; p__Campylobacterota; c__Campylobacteria; o__Campylobacterales; f__Helicobacteraceae; g__Helicobacter_D; s__Helicobacter_D sp014799995 | 51.300000 | 30.070000 | gut metagenome (*Myodes glareolus*) | Host-associated;Mammals;Digestive system;Unclassified;Unclassified | 0.173% |
|  | GCF_014799995.1 | d__Bacteria; p__Campylobacterota; c__Campylobacteria; o__Campylobacterales; f__Helicobacteraceae; g__Helicobacter_D; s__Helicobacter_D sp014799995 | 51.300000 | 30.070000 | gut metagenome (*Myodes glareolus*) | Host-associated;Mammals;Digestive system;Unclassified;Unclassified | 0.174% |
|  | GCF_014799995.1 | d__Bacteria; p__Campylobacterota; c__Campylobacteria; o__Campylobacterales; f__Helicobacteraceae; g__Helicobacter_D; s__Helicobacter_D sp014799995 | 51.300000 | 30.070000 | gut metagenome (*Myodes glareolus*) | Host-associated;Mammals;Digestive system;Unclassified;Unclassified | 0.165% |
|  | GCF_014799995.1 | d__Bacteria; p__Campylobacterota; c__Campylobacteria; o__Campylobacterales; f__Helicobacteraceae; g__Helicobacter_D; s__Helicobacter_D sp014799995 | 51.300000 | 30.070000 | gut metagenome (*Myodes glareolus*) | Host-associated;Mammals;Digestive system;Unclassified;Unclassified | 0.181% |
|  | GCF_014799995.1 | d__Bacteria; p__Campylobacterota; c__Campylobacteria; o__Campylobacterales; f__Helicobacteraceae; g__Helicobacter_D; s__Helicobacter_D sp014799995 | 51.300000 | 30.070000 | gut metagenome (*Myodes glareolus*) | Host-associated;Mammals;Digestive system;Unclassified;Unclassified | 0.180% |
|  | GCF_014799995.1 | d__Bacteria; p__Campylobacterota; c__Campylobacteria; o__Campylobacterales; f__Helicobacteraceae; g__Helicobacter_D; s__Helicobacter_D sp014799995 | 51.300000 | 30.070000 | gut metagenome (*Myodes glareolus*) | Host-associated;Mammals;Digestive system;Unclassified;Unclassified | 0.155% |
|  | GCF_014799995.1 | d__Bacteria; p__Campylobacterota; c__Campylobacteria; o__Campylobacterales; f__Helicobacteraceae; g__Helicobacter_D; s__Helicobacter_D sp014799995 | 51.300000 | 30.070000 | gut metagenome (*Myodes glareolus*) | Host-associated;Mammals;Digestive system;Unclassified;Unclassified | 0.167% |
|  | GCF_014799995.1 | d__Bacteria; p__Campylobacterota; c__Campylobacteria; o__Campylobacterales; f__Helicobacteraceae; g__Helicobacter_D; s__Helicobacter_D sp014799995 | 51.300000 | 30.070000 | gut metagenome (*Myodes glareolus*) | Host-associated;Mammals;Digestive system;Unclassified;Unclassified | 0.203% |
|  | GCF_014799995.1 | d__Bacteria; p__Campylobacterota; c__Campylobacteria; o__Campylobacterales; f__Helicobacteraceae; g__Helicobacter_D; s__Helicobacter_D sp014799995 | 51.300000 | 30.070000 | gut metagenome (*Myodes glareolus*) | Host-associated;Mammals;Digestive system;Unclassified;Unclassified | 0.184% |
|  | GCF_014799995.1 | d__Bacteria; p__Campylobacterota; c__Campylobacteria; o__Campylobacterales; f__Helicobacteraceae; g__Helicobacter_D; s__Helicobacter_D sp014799995 | 51.300000 | 30.070000 | gut metagenome (*Myodes glareolus*) | Host-associated;Mammals;Digestive system;Unclassified;Unclassified | 0.168% |
|  | GCF_014799995.1 | d__Bacteria; p__Campylobacterota; c__Campylobacteria; o__Campylobacterales; f__Helicobacteraceae; g__Helicobacter_D; s__Helicobacter_D sp014799995 | 51.300000 | 30.070000 | gut metagenome (*Myodes glareolus*) | Host-associated;Mammals;Digestive system;Unclassified;Unclassified | 0.182% |
|  | GCF_014799995.1 | d__Bacteria; p__Campylobacterota; c__Campylobacteria; o__Campylobacterales; f__Helicobacteraceae; g__Helicobacter_D; s__Helicobacter_D sp014799995 | 51.300000 | 30.070000 | gut metagenome (*Myodes glareolus*) | Host-associated;Mammals;Digestive system;Unclassified;Unclassified | 0.182% |
|  | GCF_014799995.1 | d__Bacteria; p__Campylobacterota; c__Campylobacteria; o__Campylobacterales; f__Helicobacteraceae; g__Helicobacter_D; s__Helicobacter_D sp014799995 | 51.300000 | 30.070000 | gut metagenome (*Myodes glareolus*) | Host-associated;Mammals;Digestive system;Unclassified;Unclassified | 0.175% |
|  | GCF_014799995.1 | d__Bacteria; p__Campylobacterota; c__Campylobacteria; o__Campylobacterales; f__Helicobacteraceae; g__Helicobacter_D; s__Helicobacter_D sp014799995 | 51.300000 | 30.070000 | gut metagenome (*Myodes glareolus*) | Host-associated;Mammals;Digestive system;Unclassified;Unclassified | 0.153% |
| 57 | GCF_017646085.1 | d__Bacteria; p__Campylobacterota; c__Campylobacteria; o__Campylobacterales; f__Campylobacteraceae; g__Campylobacter; s__Campylobacter sp017646085 | N/A | N/A | yak gut metagenome | Host-associated;Mammals;Digestive system;Unclassified;Unclassified | 0.013% |
|  | GCF_017646085.1 | d__Bacteria; p__Campylobacterota; c__Campylobacteria; o__Campylobacterales; f__Campylobacteraceae; g__Campylobacter; s__Campylobacter sp017646085 | N/A | N/A | yak gut metagenome | Host-associated;Mammals;Digestive system;Unclassified;Unclassified | 0.052% |
|  | GCF_017646085.1 | d__Bacteria; p__Campylobacterota; c__Campylobacteria; o__Campylobacterales; f__Campylobacteraceae; g__Campylobacter; s__Campylobacter sp017646085 | N/A | N/A | yak gut metagenome | Host-associated;Mammals;Digestive system;Unclassified;Unclassified | 0.009% |
|  | GCF_017646085.1 | d__Bacteria; p__Campylobacterota; c__Campylobacteria; o__Campylobacterales; f__Campylobacteraceae; g__Campylobacter; s__Campylobacter sp017646085 | N/A | N/A | yak gut metagenome | Host-associated;Mammals;Digestive system;Unclassified;Unclassified | 0.043% |
|  | GCF_017646085.1 | d__Bacteria; p__Campylobacterota; c__Campylobacteria; o__Campylobacterales; f__Campylobacteraceae; g__Campylobacter; s__Campylobacter sp017646085 | N/A | N/A | yak gut metagenome | Host-associated;Mammals;Digestive system;Unclassified;Unclassified | 0.029% |
| 58 | GCF_020024835.1 | d__Bacteria; p__Campylobacterota; c__Campylobacteria; o__Campylobacterales; f__Campylobacteraceae; g__Campylobacter_D; s__Campylobacter_D sp020024835 | -62.220000 | 58.790000 | gut metagenome (*Leptonychotes weddellii*) | Host-associated;Mammals;Digestive system;Unclassified;Unclassified | 1.054% |
| 59 | GCF_022511165.1 | d__Bacteria; p__Campylobacterota; c__Campylobacteria; o__Campylobacterales; f__Helicobacteraceae; g__Helicobacter_C; s__Helicobacter_C sp022511165 | 33.550000 | 97.600000 | gut metagenome (*Plateau pika*) | Host-associated;Mammals;Digestive system;Unclassified;Unclassified | 0.176% |
| 60 | GCF_022766795.1 | d__Bacteria; p__Campylobacterota; c__Campylobacteria; o__Campylobacterales; f__Helicobacteraceae; g__Helicobacter_D; s__Helicobacter_D sp022766795 | 52.468500 | -113.730700 | pig gut metagenome (*Sus scrofa domesticus*) | Host-associated;Mammals;Digestive system;Unclassified;Unclassified | 0.784% |
| 61 | GCF_029067145.1 | d__Bacteria; p__Campylobacterota; c__Campylobacteria; o__Campylobacterales; f__Helicobacteraceae; g__Helicobacter_D; s__Helicobacter_D sp029067145 | 54.690000 | 25.230000 | gut metagenome (*Apodemus flavicollis*) | Host-associated;Mammals;Digestive system;Unclassified;Unclassified | 1.736% |
| 62 | GCF_905372745.1 | d__Bacteria; p__Campylobacterota; c__Campylobacteria; o__Campylobacterales; f__Campylobacteraceae; g__Campylobacter_A; s__Campylobacter_A sp905372745 | N/A | N/A | human oral metagenome | Host-associated;Mammals: Human;Digestive system;Oral cavity;Unclassified | 2.070% |
| 63 | GCF_910577135.1 | d__Bacteria; p__Campylobacterota; c__Campylobacteria; o__Campylobacterales; f__Helicobacteraceae; g__Helicobacter_C; s__Helicobacter_C sp910577135 | 30.350000 | 114.330000 | mouse gut | Host-associated;Mammals;Digestive system;Unclassified;Unclassified | 0.975% |
| 64 | GCF_937997465.1 | d__Bacteria; p__Campylobacterota; c__Campylobacteria; o__Campylobacterales; f__Campylobacteraceae; g__Campylobacter_A; s__Campylobacter_A sp937997465 | N/A | N/A | human gut metagenome | Host-associated;Mammals: Human;Digestive system;Oral cavity;Unclassified | 0.404% |
| 65 | GCF_938018145.1 | d__Bacteria; p__Campylobacterota; c__Campylobacteria; o__Campylobacterales; f__Campylobacteraceae; g__Campylobacter_A; s__Campylobacter_A sp938018145 | N/A | N/A | human gut metagenome | Host-associated;Mammals: Human;Digestive system;Oral cavity;Unclassified | 0.629% |
| 66 | GCF_938030785.1 | d__Bacteria; p__Campylobacterota; c__Campylobacteria; o__Campylobacterales; f__Campylobacteraceae; g__Campylobacter_B; s__Campylobacter_B sp938030785 | N/A | N/A | human gut metagenome | Host-associated;Mammals: Human;Digestive system;Oral cavity;Unclassified | 0.766% |
| 67 | GCF_938039245.1 | d__Bacteria; p__Campylobacterota; c__Campylobacteria; o__Campylobacterales; f__Campylobacteraceae; g__Campylobacter_A; s__Campylobacter_A sp938039245 | N/A | N/A | human gut metagenome | Host-associated;Mammals: Human;Digestive system;Oral cavity;Unclassified | 0.513% |
| 68 | GCF_938040115.1 | d__Bacteria; p__Campylobacterota; c__Campylobacteria; o__Campylobacterales; f__Campylobacteraceae; g__Campylobacter_A; s__Campylobacter_A sp938040115 | N/A | N/A | human gut metagenome | Host-associated;Mammals: Human;Digestive system;Oral cavity;Unclassified | 0.719% |
| 69 | GCF_938045625.1 | d__Bacteria; p__Campylobacterota; c__Campylobacteria; o__Campylobacterales; f__Campylobacteraceae; g__Campylobacter_A; s__Campylobacter_A sp938045625 | N/A | N/A | human gut metagenome | Host-associated;Mammals: Human;Digestive system;Oral cavity;Unclassified | 0.662% |
| 70 | GCF_944323605.1 | d__Bacteria; p__Campylobacterota; c__Campylobacteria; o__Campylobacterales; f__Campylobacteraceae; g__Campylobacter_D; s__Campylobacter_D sp944323605 | 48.858300 | 2.292300 | chicken caecal content | Host-associated;Birds;Digestive system;Unclassified;Unclassified | 0.631% |
| 71 | GCF_947255105.1 | d__Bacteria; p__Campylobacterota; c__Campylobacteria; o__Campylobacterales; f__Campylobacteraceae; g__Campylobacter_B; s__Campylobacter_B sp947255105 | 39.290400 | -76.612200 | vaginal metagenome | Host-associated;Mammals: Human;Reproductive system;Vagina;Unclassified | 2.796% |
| 72 | GCF_947367035.1 | d__Bacteria; p__Campylobacterota; c__Campylobacteria; o__Campylobacterales; f__Helicobacteraceae; g__Helicobacter_D; s__Helicobacter_D sp947367035 | 30.400000 | 120.700000 | mouse gut metagenome | Host-associated;Mammals;Digestive system;Unclassified;Unclassified | 0.597% |
| 73 | GCA_019112865.1 | d__Bacteria; p__Campylobacterota; c__Campylobacteria; o__Campylobacterales; f__Helicobacteraceae; g__Helicobacter_F; s__Helicobacter_F avicola | N/A | N/A | gut metagenome (Gallus gallus) | Host-associated;Birds;Digestive system;Unclassified;Unclassified | 0.01762 |
| 74 | GCA_031275395.1 | d__Bacteria; p__Campylobacterota; c__Campylobacteria; o__Campylobacterales; f__SZUA-545; g__JAIRNH01; s__JAIRNH01 sp031275395 | 26.083965 | -80.239393 | termite gut (Incisitermes snyderi) | Host-associated;Arthropoda: Insects;Digestive system;Unclassified;Unclassified | 0.00388 |
| 75 | GCF_002633015.1 | d__Bacteria; p__Campylobacterota; c__Campylobacteria; o__Campylobacterales; f__Sulfurimonadaceae; g__Sulfuricurvum; s__Sulfuricurvum sp002633015 | 37.310000 | -97.440000 | water from CO2 storage reservoir | Engineered;Artificial ecosystem;Unclassified;Unclassified;Unclassified | 0.00282 |
|  | GCF_002633015.1 | d__Bacteria; p__Campylobacterota; c__Campylobacteria; o__Campylobacterales; f__Sulfurimonadaceae; g__Sulfuricurvum; s__Sulfuricurvum sp002633015 | 37.310000 | -97.440000 | water from CO2 storage reservoir | Engineered;Artificial ecosystem;Unclassified;Unclassified;Unclassified | 0.24631 |
|  | GCF_002633015.1 | d__Bacteria; p__Campylobacterota; c__Campylobacteria; o__Campylobacterales; f__Sulfurimonadaceae; g__Sulfuricurvum; s__Sulfuricurvum sp002633015 | 37.310000 | -97.440000 | water from CO2 storage reservoir | Engineered;Artificial ecosystem;Unclassified;Unclassified;Unclassified | 0.00286 |
|  | GCF_002633015.1 | d__Bacteria; p__Campylobacterota; c__Campylobacteria; o__Campylobacterales; f__Sulfurimonadaceae; g__Sulfuricurvum; s__Sulfuricurvum sp002633015 | 37.310000 | -97.440000 | water from CO2 storage reservoir | Engineered;Artificial ecosystem;Unclassified;Unclassified;Unclassified | 0.0193 |

^a^ Information not found is marked as N/A.

Table S3. Genome statistics.

|  | *Aliarcobacter* *butzleri* hDNRA1 | *Sulfurospirillum* sp. hDNRA2 |
| --- | --- | --- |
| Genome size (bp) | 2,306,496 | 2,684,720 |
| GC percentage (%) | 27.03 | 43.96 |
| CheckM completeness (%) | 100.0 | 100.0 |
| CheckM contamination (%) | 0.41 | 0.46 |
| Number of contigs | 1 | 2 (including 1 plasmid) |
| Number of genes predicted | 2,329 | 2,672 |
| Number of protein coding genes | 2,263 | 2,619 |
| Number of tRNA genes | 56 | 47 |
| Number of rRNA genes | 10 | 6 |

Table S4. List of functional genes relevant to nitrogen and hydrogen metabolisms in *Aliarcobacter* *butzleri* hDNRA1 and *Sulfurospirillum* sp. hDNRA2.

| **Gene name** | **Locus tag** | **Gene product^a^** | **Accession** | **E-value** | **EC number** | **Length (bp)** |
| --- | --- | --- | --- | --- | --- | --- |
| *Aliarcobacter* *butzleri* hDNRA1 | | | | | | |
| Nitrogen metabolism | | | | | | |
| NO_3_^−^ reduction to NO_2_^−^ | | | | | | |
| *napD* | GODOFOPP_00358 | Chaperone NapD | COG3062 | 1.52e-21 | - | 384 |
| *napL* | GODOFOPP_00359 | WD40 repeat, putative periplasmic protein | COG2319 | 1.13e-08 | - | 960 |
| *napF* | GODOFOPP_00360 | Ferredoxin-type protein NapF | PRK10194 | 5.19e-21 | - | 477 |
| *napB* | GODOFOPP_00361 | Periplasmic nitrate reductase, small subunit (cytochrome *c*-type subunit) | COG3043 | 7.68e-40 | - | 606 |
| *napH* | GODOFOPP_00362 | Ferredoxin-type protein NapH | TIGR02163 | 4.26e-107 | - | 807 |
| *napG* | GODOFOPP_00363 | Ferredoxin-type protein NapG | TIGR00397 | 7.97e-68 | - | 813 |
| *napA* | GODOFOPP_00364 | Periplasmic nitrate reductase, larges subunit | TIGR01706 | 0.0 | 1.9.6.1 | 2,811 |
| NO_2_^−^ reduction to NH_4_^+^ | | | | | | |
| *nrfA* | GODOFOPP_00353 | Cytochrome *c* nitrite reductase (ammonia forming), large subunit | PRK11125 | 0.0 | 1.7.2.2 | 1,491 |
| *nrfH* | GODOFOPP_00354 | Cytochrome *c* nitrite reductase (ammonia forming), small subunit | TIGR03153 | 1.79e-52 | - | 531 |
| N_2_O reduction to N_2_ | | | | | | |
| *nosZ* | GODOFOPP_00989 | Nitrous oxide reductase | COG4263 | 0.0 | 1.7.2.4 | 2,592 |
| *HP* | GODOFOPP_00990 | Hypothetical protein | - | - | - | 765 |
| *nosD* | GODOFOPP_00991 | Nitrous oxide reductase family maturation protein NosD | TIGR04247 | 1.54e-144 | - | 1,227 |
| *-* | GODOFOPP_00992 | Ferredoxin-type protein NapG | PRK09476 | 2.23e-48 | - | 717 |
| *-* | GODOFOPP_00993 | Cytochrome *c*_553_ | COG2863 | 4.1e-10 | - | 558 |
| *-* | GODOFOPP_00994 | Cytochrome *c*_553_ | COG2863 | 2.45e-13 | - | 444 |
| *-* | GODOFOPP_00995 | Ferredoxin-type protein NapH | PRK09477 | 5.84e-98 | - | 894 |
| *-* | GODOFOPP_00996 | Putative ABC transporter ATP-binding protein | TIGR04521 | 4.11e-34 | - | 639 |
| *nosL* | GODOFOPP_00997 | Nitrous oxide reductase accessory protein NosL | COG4314 | 3.51e-21 | - | 447 |
| *nosY* | GODOFOPP_00998 | ABC-type transport system involved in multi-copper enzyme maturation, permease component | COG1277 | 2.28e-14 | - | 828 |
| Hydrogenases^b^ | | | | | | |
| *huaS* | GODOFOPP_01010 | Group 2d [NiFe]-hydrogenase, small subunit | COG1740 | 3.52e-61 | - | 894 |
| *huaL* | GODOFOPP_01011 | Group 2d [NiFe]-hydrogenase, large subunit | COG0374 | 2.49e-71 | - | 1,350 |
| *hynA* | GODOFOPP_01012 | Group 1b [NiFe]-hydrogenase, small subunit | COG1740 | 0.0 | - | 1,236 |
| *hynB* | GODOFOPP_01013 | Group 1b [NiFe]-hydrogenase, large subunit | COG0374 | 0.0 | - | 1,743 |
| *hynC* | GODOFOPP_01014 | [NiFe]-hydrogenase cytochrome *b* subunit | COG1969 | 2.14e-64 | - | 714 |
| *hupD* | GODOFOPP_01015 | [NiFe]-hydrogenase maturation factor | COG0680 | 2.10e-32 | - | 591 |
| *hypF* | GODOFOPP_01016 | [NiFe]-hydrogenase maturation factor HypF (carbamoyltransferase) | COG0068 | 1.64e-07 | - | 1,533 |
| *hynA* | GODOFOPP_01019 | Group 1b [NiFe]-hydrogenase, small subunit | COG1740 | 0.0 | - | 1,179 |
| *hynB* | GODOFOPP_01020 | Group 1b [NiFe]-hydrogenase, large subunit | COG0374 | 0.0 | - | 1,731 |
| *hynC* | GODOFOPP_01021 | [NiFe]-hydrogenase cytochrome *b* subunit | COG1969 | 7.57e-41 | - | 666 |
| *hyaD* | GODOFOPP_01022 | [NiFe]-hydrogenase maturation factor | COG0680 | 7.30e-30 | - | 579 |
| *HP* | GODOFOPP_01023 | Hypothetical protein | - | - | - | 1,620 |
| *hypF* | GODOFOPP_01024 | [NiFe]-hydrogenase maturation factor HypF (carbamoyltransferase) | COG0068 | 0.0 | - | 2,247 |
| *hypB* | GODOFOPP_01048 | Hydrogenase/urease maturation factor HypB, Ni^2+^-binding GTPase | COG0378 | 1.55e-97 | - | 816 |
| *hypC* | GODOFOPP_01049 | Hydrogenase maturation factor HybG, HypC/HupF family | COG0298 | 1.40e-29 | - | 282 |
| *hypD* | GODOFOPP_01050 | Hydrogenase maturation factor HypD | COG0409 | 0.0 | - | 1,125 |
| *hypE* | GODOFOPP_01055 | Carbamoyl dehydratase HypE (hydrogenase maturation factor) | COG0309 | 3.71e-162 | - | 999 |
| *hypA* | GODOFOPP_01056 | Hydrogenase maturation factor HypA/HybF, metallochaperone involved in Ni insertion | COG0375 | 2.77e-39 | - | 342 |
| *Sulfurospirillum* sp. hDNRA2 | | | | | | |
| Nitrogen metabolism | | | | | | |
| NO_3_^−^ reduction to NO_2_^−^ | | | | | | |
| *napD* | NBADHHHO_01834 | Chaperone NapD | COG3062 | 2.32e-23 | - | 354 |
| *napL* | NBADHHHO_01835 | WD40 repeat, putative periplasmic protein | COG2319 | 4.86e-18 | - | 948 |
| *napF* | NBADHHHO_01836 | Ferredoxin-type protein NapF | PRK10194 | 4.16e-24 | - | 504 |
| *napB* | NBADHHHO_01837 | Periplasmic nitrate reductase, small subunit (cytochrome *c*-type subunit) | COG3043 | 5.41e-54 | - | 525 |
| *napH* | NBADHHHO_01838 | Ferredoxin-type protein NapH | TIGR02163 | 2.29e-113 | - | 825 |
| *napG* | NBADHHHO_01839 | Ferredoxin-type protein NapG | TIGR00397 | 1.96e-62 | - | 822 |
| *napA* | NBADHHHO_01840 | Periplasmic nitrate reductase catalytic/large subunit | TIGR01706 | 0.0 | 1.9.6.1 | 2,790 |
| NO_2_^−^ reduction to NH_4_^+^ | | | | | | |
| *nrfA* | NBADHHHO_01869 | Cytochrome *c* nitrite reductase (ammonia forming), large subunit | PRK11125 | 0.0 | 1.7.2.2 | 1,548 |
| *nrfH* | NBADHHHO_01870 | Cytochrome *c* nitrite reductase (ammonia forming), small subunit | TIGR03153 | 4.11e-62 | - | 534 |
| N_2_O reduction to N_2_ | | | | | | |
| *nosZ* | NBADHHHO_01560 | Nitrous-oxide reductase | COG4263 | 0.0 | 1.7.2.4 | 2,595 |
| *nosY* | NBADHHHO_01747 | ABC-type transport system involved in multi-copper enzyme maturation, permease component | COG1277 | 5.57e-15 | - | 828 |
| *nosL* | NBADHHHO_01748 | Nitrous oxide reductase accessory protein NosL | COG4314 | 3.78e-11 | - | 504 |
| *HP* | NBADHHHO_01749 | Hypothetical protein | - | - | - | 966 |
| *lolD* | NBADHHHO_01750 | ABC-type lipoprotein export system, ATPase component | COG1136 | 1.10e-95 | - | 669 |
| *lolC* | NBADHHHO_01751 | ABC-type transport system involved in lipoprotein release, permease component | COG4591 | 2.45e-27 | - | 1,194 |
| *HP* | NBADHHHO_01752 | Hypothetical protein | - | - | - | 318 |
| *nosL* | NBADHHHO_01753 | Nitrous oxide reductase accessory protein NosL | COG4314 | 2.62e-22 | - | 456 |
| *-* | NBADHHHO_01754 | Putative ABC transporter ATP-binding protein | pfam00005 | 1.09e-31 | - | 639 |
| *-* | NBADHHHO_01755 | Ferredoxin-type protein NapH | TIGR02163 | 6.19e-91 | - | 906 |
| *-* | NBADHHHO_01756 | Cytochrome *c*_553_ | COG2863 | 3.56e-16 | - | 495 |
| *-* | NBADHHHO_01757 | Cytochrome *c*_553_ | COG2863 | 9.39e-13 | - | 579 |
| *-* | NBADHHHO_01758 | Ferredoxin-type protein NapG | TIGR00397 | 1.82e-32 | - | 726 |
| *nosD* | NBADHHHO_01759 | Nitrous oxide reductase family maturation protein NosD | TIGR04247 | 1.38e-151 | - | 1,308 |
| *HP* | NBADHHHO_01760 | Hypothetical protein | - | - | - | 795 |
| *nosZ* | NBADHHHO_01761 | Nitrous-oxide reductase | COG4263 | 0.0 | 1.7.2.4 | 2,601 |
| Hydrogenase | | | | | | |
| *hypA* | NBADHHHO_01203 | Hydrogenase maturation factor HypA/HybF, metallochaperone involved in Ni insertion | COG0375 | 4.10e-42 | - | 342 |
| *hypE* | NBADHHHO_01204 | Carbamoyl dehydratase HypE (hydrogenase maturation factor) | COG0309 | 1.62e-166 | - | 1,005 |
| *hypD* | NBADHHHO_01205 | Hydrogenase maturation factor HypD | COG0409 | 0.0 | - | 1,125 |
| *hypC* | NBADHHHO_01206 | Hydrogenase maturation factor HybG, HypC/HupF family | COG0298 | 2.00e-32 | - | 270 |
| *hypB* | NBADHHHO_01207 | Hydrogenase/urease maturation factor HypB, Ni^2+^-binding GTPase | COG0378 | 1.21e-124 | - | 837 |
| *hypF* | NBADHHHO_01382 | [NiFe]-hydrogenase maturation factor HypF (carbamoyltransferase) | COG0068 | 0.0 | - | 2,220 |
| *HP* | NBADHHHO_01383 | Hypothetical protein | - | - | - | 1,638 |
| *hupD* | NBADHHHO_01384 | [NiFe]-hydrogenase maturation factor | COG0680 | 3.00e-32 | - | 540 |
| *hynC* | NBADHHHO_01385 | [NiFe]-hydrogenase cytochrome *b* subunit | COG1969 | 7.71e-38 | - | 678 |
| *hynB* | NBADHHHO_01386 | Group 1b [NiFe]-hydrogenase, large subunit | COG0374 | 0.0 | - | 1,749 |
| *hynA* | NBADHHHO_01387 | Group 1b [NiFe]-hydrogenase, small subunit | COG1740 | 0.0 | - | 1,158 |
| *huaL* | NBADHHHO_01388 | Group 2d [NiFe]-hydrogenase, large subunit | COG0374 | 2.05e-71 | - | 1,344 |
| *huaS* | NBADHHHO_01389 | Group 2d [NiFe]-hydrogenase, small subunit | COG1740 | 5.81e-57 | - | 897 |
| *hycG* | NBADHHHO_01479 | Group 4c [NiFe]-hydrogenase, small subunit | COG3260 | 4.87e-51 | - | 411 |
| *-* | NBADHHHO_01480 | Formate hydrogenlyase complex iron-sulfur subunit | PRK12387 | 7.08e-16 | - | 426 |
| *hycE1* | NBADHHHO_01481 | NADH-quinone oxidoreductase subunit C | COG3262 | 2.05e-17 | - | 495 |
| *hycE2* | NBADHHHO_01482 | Group 4c [NiFe]-hydrogenase, large subunit | COG3261 | 1.31e-161 | - | 1,089 |
| *hycB* | NBADHHHO_01483 | Fe-S-cluster-containing hydrogenase component 2 | cd10554 | 2.41e-55 | - | 474 |
| *hypA* | NBADHHHO_01484 | Hydrogenase maturation factor HypA/HybF, metallochaperone involved in Ni insertion | COG0375 | 5.53e-38 | - | 363 |
| *gltD* | NBADHHHO_01485 | Glutamate synthase (NADPH), beta chain | COG0493 | 0.0 | 1.4.1.13 | 1,425 |
| *-* | NBADHHHO_01844 | Putative Fe-S cluster-containing hydrogenase component | COG1142 | 6.55e-44 | - | 654 |
| *hyfB* | NBADHHHO_01845 | Putative multi-subunit Na^+^/H^+^ antiporter | COG0651 | 6.05e-86 | - | 1,941 |
| *hyfC* | NBADHHHO_01846 | Formate hydrogenlyase subunit HyfC | COG0650 | 3.97e-80 | - | 927 |
| *hyfE* | NBADHHHO_01847 | Hydrogenase membrane subunit HyfE | COG4237 | 2.73e-48 | - | 654 |
| *hyfB* | NBADHHHO_01848 | Putative multi-subunit Na^+^/H^+^ antiporter | COG0651 | 2.05e-93 | - | 1,470 |
| *hyfE* | NBADHHHO_01849 | Group 4a [NiFe]-hydrogenase large subunit | COG3261 | 0.0 | - | 1,740 |
| *-* | NBADHHHO_01850 | Formate hydrogenlyase complex iron-sulfur subunit | PRK12387 | 5.83e-98 | - | 540 |
| *hyfG* | NBADHHHO_01851 | Group 4a [NiFe]-hydrogenase small subunit | COG3260 | 1.90e-88 | - | 822 |
| *hyfH* | NBADHHHO_01852 | Formate hydrogenlyase maturation protein | PRK15084 | 5.97e-09 | - | 360 |
| *hyfD* | NBADHHHO_01853 | Endopeptidases belonging to membrane-bound hydrogen evolving hydrogenase group | cd06067 | 1.99e-45 | - | 447 |

^a^ Gene annotation was performed using NCBI’s non-redundant sequence database as the reference database.

^b^ Hydrogenase classification was in accordance with HydDB (https://services.birc.au.dk/hyddb/).

Table S5. List of genes in the aspartate ammonia-lyase cluster.

| **Gene name** | **Locus tag** | **Gene product^a^** | **Accession** | **E-value** | **EC number** | **Length (bp)** |
| --- | --- | --- | --- | --- | --- | --- |
| *hydF* | NBADHHHO_01802 | [FeFe]-hydrogenase H-cluster maturation GTPase HydF | TIGR03918 | 0.0 | - | 1,227 |
| *hydE* | NBADHHHO_01803 | [FeFe]-hydrogenase H-cluster radical SAM maturase HydE | TIGR03956 | 1.87e-127 | - | 1,068 |
| *aspA* | NBADHHHO_01804 | Aspartate ammonia-lyase | COG1027 | 0.0 | 4.3.1.1 | 1,392 |
| *hydG* | NBADHHHO_01805 | [FeFe]-hydrogenase H-cluster radical SAM maturase HydG | TIGR03955 | 2.03e-156 | - | 1473 |
| *-* | NBADHHHO_01806 | Cytochrome *b* subunit | COG2864 | 1.10e-22 | - | 690 |
| *hydB* | NBADHHHO_01807 | Group A [FeFe]-hydrogenase, small subunit | pfam02256 | 2.41e-11 | - | 363 |
| *hydA* | NBADHHHO_01808 | Group A [FeFe]-hydrogenase, large subunit | TIGR02512 | 8.78e-138 | - | 1,353 |
| 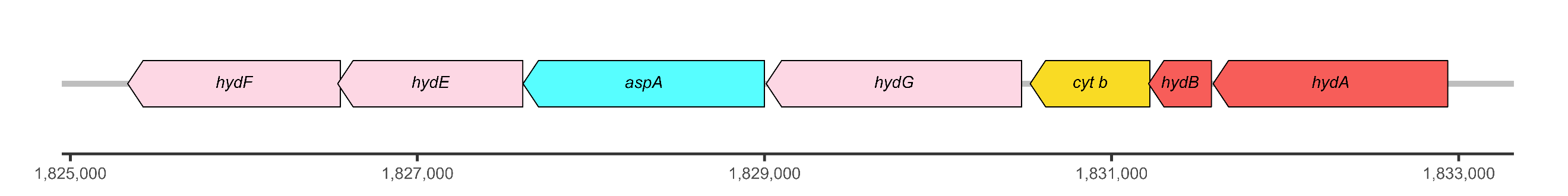 | | | | | | |

^a^ Gene annotation was performed using NCBI’s non-redundant sequence database as the reference database.
